# The spatial distribution of eye movements predicts the (false) recognition of emotional facial expressions

**DOI:** 10.1371/journal.pone.0245777

**Published:** 2021-01-26

**Authors:** Fanny Poncet, Robert Soussignan, Margaux Jaffiol, Baptiste Gaudelus, Arnaud Leleu, Caroline Demily, Nicolas Franck, Jean-Yves Baudouin

**Affiliations:** 1 Developmental Ethology and Cognitive Psychology Lab, Centre des Sciences du Goût et de l’Alimentation, AgroSup Dijon, CNRS, Inrae, Université Bourgogne Franche-Comté, Dijon, France; 2 Centre Ressource de Réhabilitation Psychosociale et de Remédiation Cognitive, Centre Hospitalier Le Vinatier & Université Lyon 1 (CNRS UMR 5229), Université de Lyon, Lyon, France; 3 Reference Center for Rare Diseases with Psychiatric Phenotype GénoPsy, Centre Hospitalier le Vinatier, Marc Jeannerod Institute (CNRS & Claude Bernard Lyon 1 University), Bron, France; 4 Laboratoire Développement, Individu, Processus, Handicap, Éducation (DIPHE), Département Psychologie du Développement, de l'Éducation et des Vulnérabilités (PsyDEV), Institut de Psychologie, Université de Lyon (Lumière Lyon 2), Lyon, France; Bournemouth University, UNITED KINGDOM

## Abstract

Recognizing facial expressions of emotions is a fundamental ability for adaptation to the social environment. To date, it remains unclear whether the spatial distribution of eye movements predicts accurate recognition or, on the contrary, confusion in the recognition of facial emotions. In the present study, we asked participants to recognize facial emotions while monitoring their gaze behavior using eye-tracking technology. In Experiment 1a, 40 participants (20 women) performed a classic facial emotion recognition task with a 5-choice procedure (anger, disgust, fear, happiness, sadness). In Experiment 1b, a second group of 40 participants (20 women) was exposed to the same materials and procedure except that they were instructed to say whether (i.e., Yes/No response) the face expressed a specific emotion (e.g., anger), with the five emotion categories tested in distinct blocks. In Experiment 2, two groups of 32 participants performed the same task as in Experiment 1a while exposed to partial facial expressions composed of actions units (AUs) present or absent in some parts of the face (top, middle, or bottom). The coding of the AUs produced by the models showed complex facial configurations for most emotional expressions, with several AUs in common. Eye-tracking data indicated that relevant facial actions were actively gazed at by the decoders during both accurate recognition and errors. False recognition was mainly associated with the additional visual exploration of less relevant facial actions in regions containing ambiguous AUs or AUs relevant to other emotional expressions. Finally, the recognition of facial emotions from partial expressions showed that no single facial actions were necessary to effectively communicate an emotional state. In contrast, the recognition of facial emotions relied on the integration of a complex set of facial cues.

## Introduction

Human facial expressions communicate various types of information about emotional states, social motives and intentions [1]. Despite the rich literature on emotion recognition testifying that discrete patterns of facial actions communicate distinct basic emotional states across different cultures (thus supporting the universality hypothesis) [1–4], recent evidence suggests a more complex pattern [4–7]. Indeed, similar facial actions may communicate different emotions or intentions based on the cultural background of the decoders; for example, a gasping face that signals “fear” in occidental populations is perceived as threatening in a Malesian isolated society [5]. Other cultural differences were reported both in the facial actions that constitute typical expressions of emotions and in the temporal dynamics of these actions [6]. In this last study, while Westerner participants represent the different fundamental emotions with distinct facial actions, Eastern participants showed some overlap between certain emotion categories, such as anger, disgust, fear and surprise. In addition, despite the use of distinct facial actions for these last emotional categories, Westerners showed less clear-cut categorical boundaries between these categories than between others [8], with frequent confusions between, for example, anger and disgust or fear and surprise [9]. For these reasons, becoming an efficient decoder of facial expressions, with a particular cultural background, is a challenge during both infancy and childhood. Becoming able to recognize the full range of facial emotions is a learning process that lasts until late childhood or even adolescence [10]. Therefore, the adult’s ability is the end product of a developmental process which relies on the simultaneous acquisition of cognitive and linguistic abilities [11, 12]. Furthermore, an impaired ability to recognize facial emotions has been associated with a broad range of neurological or psychiatric diseases [13–17], which suggests that distinct cognitive functions (e.g., attention, ability to process complex stimuli [13], and verbal skills [18, 19]) strongly participate in the ability to decode facial emotions.

Thus, recognizing facial emotions is a more complex task despite the promptness and accuracy with which we carry it out everyday. In everyday life, the distinct patterns of facial actions presumed to communicate discrete emotional states [20] are embedded in complex configurations [21]. Each basic emotional state may be communicated by several facial actions, displayed either alone or in various combinations: for example, anger can be signaled by frowning the eyebrows, gritting the teeth, tightening the lips, or any combinations of these actions. Further, according to the source of the aversive reaction (e.g., olfactory or gustatory, related to food or to physical or moral violations) [22], disgust may be conveyed either by wrinkling the nose, signaling the blocking of incoming airflow, or by opening the mouth, simulating food rejection, or by lowering eyebrows, distancing from the disgusting situation. At the same time, a single facial expression may combine two or more basic emotion categories [23], and a single facial action may be involved in different emotions (e.g., lowering eyebrows in anger, disgust, fear, or sadness). Finally, some facial actions do not have clear circumscribed consequences on face morphology. For instance, nose wrinkling may also pull down the inner part of the eyebrows (or give this visual feeling), as in frowning. Consequently, despite the involvement of discrete sets of muscles, it may be difficult to separate two facial actions based on visual inspection.

To date, despite the complexity of facial emotion recognition, it was shown that humans are able to quickly recognize facial expressions in few fixations, with the first fixation(s) made just below the eyes. This strategy optimizes the visual availability of the facial features in the foveal area [e.g., 24] and allows centralizing the main facial features before exploring the face [25–27]. This can allow to apprehend the face as a whole, and to detect areas where facial actions occur for further exploration. In this respect, it has been suggested that the first fixations allow a holistic facial processing [e.g., 27]. The information then extracted allows a first global grasp of the expression, as suggested by studies that superimposed the high spatial frequencies of a neutral face on the low frequencies of an expressive face (anger, fear, happiness, or sadness): they showed that the response is oriented towards the expression in low spatial frequencies [28–30]. Literature also agrees that some facial areas are more important for the correct recognition of emotional facial expressions [e.g., 31–34], with different regions according to the expression. While for most emotions, the eyes/eyebrows (upper part) face area is fixated longer than the mouth area (bottom part) [35], the expression of happiness presents a particular status. Indeed, happiness is always better recognized than the other expressions [e.g., 31], and it is the only expression for which the mouth is proportionally looked longer than other features and appears both sufficient and necessary for correct recognition of happiness [31, 36]. Moreover, the influence of the smiling mouth to interpret a face as happy is efficient even when the mouth is not fixated and not in the fovea [33, 34]. Nevertheless, this is the only expression for which the importance of one specific area is so clear: the recognition of other expressions seems to rely more on a combination of several facial actions located in distinct areas. Anger and fear recognition could rely more on the processing of the eyes/eyebrows region [31, 36–39]. However, this area does not appear more attractive in the processing of anger. For fear, some studies also found a crucial importance of the mouth, suggesting a possible holistic processing for this particular expression [31]. For disgust, the mouth also appears to be an important feature to recognize it [31, 32, 36–39] but could not be the only important feature [31]. The nose area could notably be attractive and of importance to process and correctly recognize disgust. The correct recognition of sadness could depend both on the eyes/eyebrows and the mouth areas, in a more balanced way [32, 36–39] with a potential advantage of the eyes/eyebrows region [31].

While these results inform about attractive power, necessity, and sufficiency of some facial areas for the correct recognition of emotional facial expressions, they do not fully inform about the precise exploratory visual patterns that allow the correct recognition of emotions. In most studies, the regions of interest included large facial areas, frequently including several features (e.g., eyes and eyebrows), and did not allow to exactly determine where the gaze landed when the participants accurately decoded the expression. Moreover, while the link between sufficiency/necessity of exploring some facial areas has already been studied in the literature [e.g., 31], the link between visual exploration and misinterpretations has, to our knowledge, not been explored yet. However, these misinterpretations are a core feature of a broad range of psychiatric disorders [40–43]. Their study in typical/healthy population, at first, would enhance the comprehension of the link between (non-adapted) visual exploratory pattern and misinterpretations of the emotional content of a face. More specifically, the reason why some participants miss the correct emotion is largely unknown. They could not gaze at the relevant facial area, or they could gaze at it and misinterpreted the facial actions. They could also gazed at areas that are not relevant for the actual emotion, but that contain facial actions that evoke other emotions, driving the participant to respond accordingly, whether or not they gaze at more relevant actions as well.

The purpose of the present study was to extend our understanding of the relationship between the facial actions used by the emitters to communicate emotions and the actual visual perception of the adult decoders. We specifically investigated, using an eye-tracking technique, the visual exploration patterns of distinct facial configurations during the decoding of emotional expressions, with a particular interest in the visual patterns that triggered misinterpretations of facial cues. We also developed a novel approach that allows us to more precisely define the location of gazes compared to the classical method that involved the defining of areas of interest (AOIs); in the present study, the gaze locations for the different emotional expressions were transformed to fit a single prototypical neutral model (see S1 Fig for an illustration). Then, we were able to contrast precise gaze locations across emotional expressions by considering the exact facial regions on which the gaze landed. Firstly, we used this approach to evidence the gaze patterns associated with correct and incorrect facial emotion recognition when participants were involved in a 5-choice task (Experiment 1a) or when they were told to say if the face expressed or not a specific emotion (e.g., “anger”; Experiment 1b). The requisite of facial actions was investigated in a second time, by displayed participants with partial expressions that comprised (or not) facial actions at different facial areas (at top, middle or bottom; Experiment 2).

## Experiments 1

In Experiment 1, the gaze behavior of participants was monitored while they had to recognize facial expressions (anger, disgust, fear, joy and sadness). The pictures came from the Karolinska Directed Emotional Faces (KDEF) database [44], and facial actions produced by the models were coded according to the FACS [20]. During the experiment, two photographs depicting the same neutral person were displayed side-by-side, with a red cross between them (see Fig 2A for an illustration). When participants had gazed at the cross for 1 sec, one of the faces changed to an expressive face (either angry, disgusted, fearful, happy, or sad), approximating the dynamic facial aspect of expressions. They were given two different instructions. In Experiment 1a, the participants performed a classic facial emotion recognition task with a 5-choice procedure (anger, disgust, fear, happiness, sadness). In Experiment 1b, they were instructed to say whether (i.e., Yes/No response) the face expressed a specific emotion (e.g., anger), in order to define the gaze pattern applied when participants search for this specific emotion. The participants were free to explore the face, and they were instructed to press the corresponding key on the keyboard as soon as they recognized one of the five emotion categories (Experiment 1a) or decided if yes or no the face expressed the target emotion (Experiment 1b).

### Methods

#### Participants

Forty participants [20 females, mean age = 25.3 ± 5.2 (SD) years, range 20.4–37.3 years; 20 males, mean age = 24.4 ± 4.6 (SD) years, range 18.8–34.6] were included in Experiment 1a, and forty new participants [20 females, mean age = 27.1 ± 6.3 (SD) years, range 18.0–38.6 years; 20 males, mean age = 25.9 ± 6.2 (SD) years, range 18.1–39.0] were included in the Experiment 1b. Fourteen additional participants participated in the experiment, but were excluded due to calibration difficulties (exceeding 1.5° of error; 7 in each experiment). All participants reported normal or corrected-to-normal vision, and no participant reported a history of psychiatric or neurological disorders. The participants provided written informed consent prior to the experiment and received financial compensation. Testing was conducted in accordance with the Declaration of Helsinki and approved by the French ethics committee (CPP Sud-Est III—2016-A02056-45).

#### Materials

We used photographs of 60 models (30 females) from the KDEF database [44]. The pictures were the same for both the 5-choice (Experiment 1a) and Yes/No (Experiment 1b) tasks. The models depicted in KDEF were amateur actors instructed to train for 1 hour before the photo session, with the purpose of evoking the emotion they were trying to express, and having the expression being strong and clear, in a way that felt natural to them. These individuals have been photographed to create this free-access images database and thus consented that their image is freely used: https://kdef.se/home/using%20and%20publishing%20kdef%20and%20akdef.html. The female models were F01-F07, F09-F13, F15, and F19-F35. The male models were M01-M02, M04-M14, M16-M18, M21-M25, and M27-M35. Six other models (F14, F16, F17, M03, M15, and M26) were used for the training sessions. For each model, we selected 6 pictures, one neutral and one for each emotion category we tested (anger, disgust, fear, happiness, sadness). The pictures were resized to 900x750 pixels from the top of the skull to the middle of the neck, with face-size adjusted to around 20 × 15 cm (presented at an approximate viewing distance of 60 cm, with an 18.9 × 14.3° of visual angle). The pictures with the emotional expressions were adjusted to superpose the main expressive features with the corresponding features in the neutral face, with the purpose of approximating a visual impression of a dynamic expression when the neutral face was replaced by an expressive face.

#### Procedure

Following the explanations about the purpose of the study, the precise task they will have to perform and signature of consent forms, the participants sat in front of the monitor and were instructed to put their head on a chin-rest at approximately 60 cm from the screen. The pictures were displayed on a 1680 x 1050 pixels screen with the Experiment Center software (version 3.4, SensoMotoricInstruments (SMI) GmbH, Teltow, Germany), designed for the SMI eye-movement tracking system (RED250, SMI). Two-dimensional recordings of the infants’ eyes were performed using the iVewX Software (version 2.4, SMI), with a sampling rate set at 250 Hz. A thirteen-point calibration was performed. The emotion recognition phase was performed after the calibration was successful (i.e., the target ideal calibration was a less than 1° lag in the x and y dimensions). During the recognition phase, each trial started with two neutral faces of the same model on each side of a red cross, which was displayed at the center of the screen. The participants were told to gaze at the red cross for 1 sec to trigger an emotional expression. After 1 sec, the left or right neutral face was replaced by an expressive face of the same model.

The instructions and exact design varied across the experiments.

*Experiment 1a*. *5-choice task*. The participants had to identify, as quickly as possible, which of the 5 emotions (anger, disgust, fear, happiness, or sadness) was mimicked by the face and to click on the corresponding key on the pad, followed by pressing the space bar (which validates their response) to move to the following trial. Participants could not correct their answer; if they clicked on several keys, only the first one was considered for analyzing. After a training session of 30 trials (6 identities (3 females/3 males) x 5 expressions), they completed 300 trials (60 identities (30 females/30 males) x 5 expressions), organized in 5 blocks of 60 trials. Each model appeared only one time in a block, and the frequencies of the 5 different expressions and of the left/right position of the emotion were the same within and between the blocks. The order of the blocks and left/right positions was alternated between participants.

*Experiment 1b*. *Yes/No task*. The participants had to decide whether the emotion expressed was the one indicated on the top of the screen (either anger, disgust, fear, happiness, or sadness) by pressing the pad-location corresponding to “Yes” or “No”. Then, they had to validate their response using the space bar. They performed the same 5 blocks as in experiment 1, with one block associated with each emotion category. The blocks were the same as in experiment 1, i.e., each emotion category occurred the same number of times within the block (12 times on 60 trials by blocks). The order of blocks as well as the emotion category associated with each block was alternated between participants. This time, a training session of 30 trials was performed before each block, with the same design as the training session for experiment 1, except for the response (Yes/No).

#### Data analyses

*Efficiency of facial expression recognition*. The dependent variables were the percentages of correct responses and, for correct responses, the number of fixations and latency from the end of the last fixation to the response. For the number of fixations and latency, we excluded trials above or below two standard deviations from the mean of the experimental condition. For all these variables, we applied three-way ANOVAs with *Facial emotion* (anger vs. disgust vs. fear vs. happiness vs. sadness) and *Gender of the model* (female vs. male) as within-subject factors and *Gender of the decoder* (female vs. male) as a between-subjects factor. Indeed, since some recent studies have not always found a difference of recognition performance between women and men [45], previous reviews and meta-analyses revealed a small to moderate female advantage [46–48], encouraging us to verify a possible influence of the gender of the observer/decoder. For all ANOVAs, we used the Greenhouse-Geisser epsilon adjustment. For the main effect of *Facial emotion*, post hoc comparisons were performed using Tukey’s tests. For interactions involving the factors *Gender of the model* and *Facial emotion*, we performed linear contrasts to test for the effect of gender for each expression, using the Holm-Bonferroni method to correct for multiple comparisons.

*Coding of facial actions*. The facial expressions produced by the different models were coded by a trained FACS coder (RS). For each picture, he listed the facial AUs used by the model to simulate the target emotional state, without being aware of the emotion the model attempted to simulate. Then, for each emotion, we computed the frequency of the different AUs in the 60 models. The participants’ efficiency in the recognition of mimicked facial emotions was analyzed by computing nonparametric Mann-Whitney tests to compare accuracy (percentage of correct recognition) as well as the number of fixations and latencies from stimulus display to the end of last fixation for the response in correct recognition trials, when the facial action was present vs. absent in the models.

*Oculometric data transformation*. The fixation coordinates were transformed in such a way that the locations of the gaze on the original facial pictures were relocated to match the same locations on a prototypical picture (i.e., that correspond to the average of all neutral pictures used in the course of the experiment, see S1 Fig). To do that, we measured the coordinates (x,y) of 73 points on each photograph. The same procedure was applied to prototypical faces. The 73 points of a picture served to define 130 triangles, which covered the internal facial features. Thus, each fixation (F) on the original picture filled in one triangle ABC. From the coordinates of this triangle, we computed two parameters: the angles formed by BAC and BAF, and the distances between A and F and between A and the projection of F (F’) on the line BC. Then, the ratio of the two angles (ABF/ABC) and the ratio of the two distances (AF/AF’) were applied to the same triangle in the prototypical face to compute the transformed coordinates (f”) of the fixation.

The second step was to compute an area around each fixation that simulated the acuity within the foveal zone, i.e., the area of the face that was best perceived. For that purpose, we defined an area of 96 pixels around the fixations (approximately 1° radius of visual angle, which is the approximate size of the foveal zone). A cumulative density function for normal distributions was then applied to pixels surrounding the fixation, with an asymptote at the fixation location that corresponded to the fixation’s duration. The mean of this cumulative function corresponded to 96 pixels (before transformation) and the standard deviation of 25% of the mean (24 pixels before transformation). With this procedure, the duration computed for the pixels neighboring the fixation (under 1° of visual angle) was close to the duration of fixation and decreased abruptly at approximately 1°. An illustration is displayed in S1 Fig. Then, we could average the fixations’ durations for different faces and participants, as illustrated in Fig 2B and 2C. These figures illustrate the time the different areas of the face were within the foveal zone of the participants. This approach allowed us to perform analyses on the exact location of fixations, avoiding the approach used with an AOI analysis and its limitations (i.e., difficulties in defining the appropriate number, size, shape and precise borders of AOIs). We obtained the “exploratory maps” (displayed in the figures) that represent the exploration patterns for the different expressions and allowed “pixel by pixel” statistical analyses.

*Oculometric data analyses*. To investigate whether the participants’ visual exploratory patterns for the different facial regions varied according to the type of emotional expression and the accuracy of the responses (correct vs. incorrect), we computed a series of Student’s T-tests for each pixel. First, for each pixel of a given picture (e.g., model F01 expressing anger), we averaged the fixation durations of the 40 participants who performed the task. Then, for each pixel again, we averaged the values obtained for the 60 models, which corresponded to the mean time the pixel was within the foveal zone. These values served to create the “Time” illustrations (displayed in the figures) after a transformation to color codes. We also computed the standard deviations between the models to further compute Student’s T-tests.

Then, a series of Student’s T-tests (with models as aleatory factor) was computed for each pixel to investigate two types of differences: (i) between emotions and (ii) between correct vs. incorrect responses. For the first type of differences, the mean time on the pixel for a given emotion (e.g., anger) was contrasted with the mean time on the corresponding pixel averaged across the 4 other emotions (i.e., disgust, fear, happiness, and sadness). For the second type of difference, the mean time for errors was contrasted with the mean time for correct responses, as illustrated in Fig 2. Considering the number of pixels (750x900), no correction was performed. However, only regions with *p* values < .0001 are discussed throughout the manuscript.

### Results

#### Accuracy in recognizing facial emotions

To investigate the participants’ accuracy in recognizing facial emotions in the 5-choice and the Yes/No tasks, we performed six (i.e., three per experiment) 5x2x2 ANOVAs with Facial emotion (anger vs. disgust vs. fear vs. happiness vs. sadness) and Gender of model (female vs. male) as the within-subject factors, and Gender of the decoder (female vs. male) as the between-subjects factor, on the percentages of correct recognition as well as, for correct responses, the number of fixations and the latency from stimulus display to the end of the last fixation before the response (see S3 Fig for details of the results). Since the results were similar in both experiments, we report them together.

These analyses revealed a significant main effect of Facial emotion for all the indicators in the two experiments (lower F value: F(4,152) = 15.71, Ɛ = .87, η_p_^2^ = .29, p < .0001). In both experiments, the participants were more accurate in recognizing happiness than any other emotion, and they made fewer fixations and were quicker to make the correct response (i.e., latency from the end of the last fixation; all *p* < .0009 after post hoc HSD of Tukey tests). Additional ANOVAs restricted to the other expressions (i.e., after removing happiness from the factor Facial emotion) further showed that the main effect of Facial emotion was still significant for all indicators (lower F value: F(3,114) = 2.89, Ɛ = .92, η_p_^2^ = .07, p = .00436), with accuracy (percentage of correct responses, number of fixations, and/or latency to the end of the last fixation) being higher for anger and lower for fear. The main effect of Gender of the model was also significant for several indicators, but this effect depended on the particular facial emotions: overall, the participants were more accurate (i.e., higher percentage of correct recognition and/or fewer fixations and/or shorter latencies) with male than female models mimicking anger and disgust, and the difference reached significance after post hoc HSD of Tukey tests in the 5-choice task on percentage for anger (p = .0019) and on the number of fixations for disgust (p = .0054). The Gender of the decoder had no effect, either alone or in interaction with other factors.

Note that accuracy was similar for the two tasks (5-choice: M = 86.9, SD = 4.6; Yes/No: M = 86.8, SD = 5.7; T(78) = .11). Furthermore, despite fewer fixations and shorter latencies in the Yes/No task than in the 5-choice task, the differences were not significant for the number of fixations (5-choice: M = 3.3, SD = 1.3; Yes/No: M = 3, SD = 0.7; T(78) = 1.33) or for the latencies of correct responses (5-choice: M = 1290 ms, SD = 445; Yes/No: M = 1229, SD = 266; T(78) = .75).

The analyses of false recognitions in the 5-choice task (i.e., an emotion was perceived in a face that mimicked another emotion) also revealed that some emotions were more frequently falsely recognized than others (see S4 Fig). That is, a 5x2x2 ANOVA with the factors Facial emotion (anger vs. disgust vs. fear vs. happiness vs. sadness; within-subjects), Gender of model (female vs. male; within-subjects) and Gender of decoder (female vs. male; between-subjects) revealed a main effect of Facial emotion (F(4,152) = 24.38, Ɛ = .66, η_p_^2^ = .39, p < .0001) (see S4A Fig). Recognizing disgust or sadness in other expressions was significantly more frequent (5.6% and 4.9% of responses, respectively) than anger and fear (2.7% and 2.6%, respectively); very few false perceptions of happiness occurred (0.4%) (all p*s* < .0046 among these three groups after HSD of Tukey tests). Gender of model interacted with this effect (F(4,152) = 3.95, Ɛ = .77, η_p_^2^ = .09, p = .0094), with a higher level of false recognition of fear with the female models than with the male models (3.2% vs. 1.9%, p = .0131 after HSD of Tukey tests). Looking at the errors for the different emotions (S4B Fig) further showed that anger was more frequently mistaken for disgust, fear for sadness, and sadness for fear or disgust, whereas disgust was confounded quite equally with all other negative emotions. Note that the Gender of the decoders had no effect either alone or in interaction with the other factors. Thus, we did not consider this factor anymore.

#### The facial actions that drove accurate recognition of facial emotion

**Fig 1:**
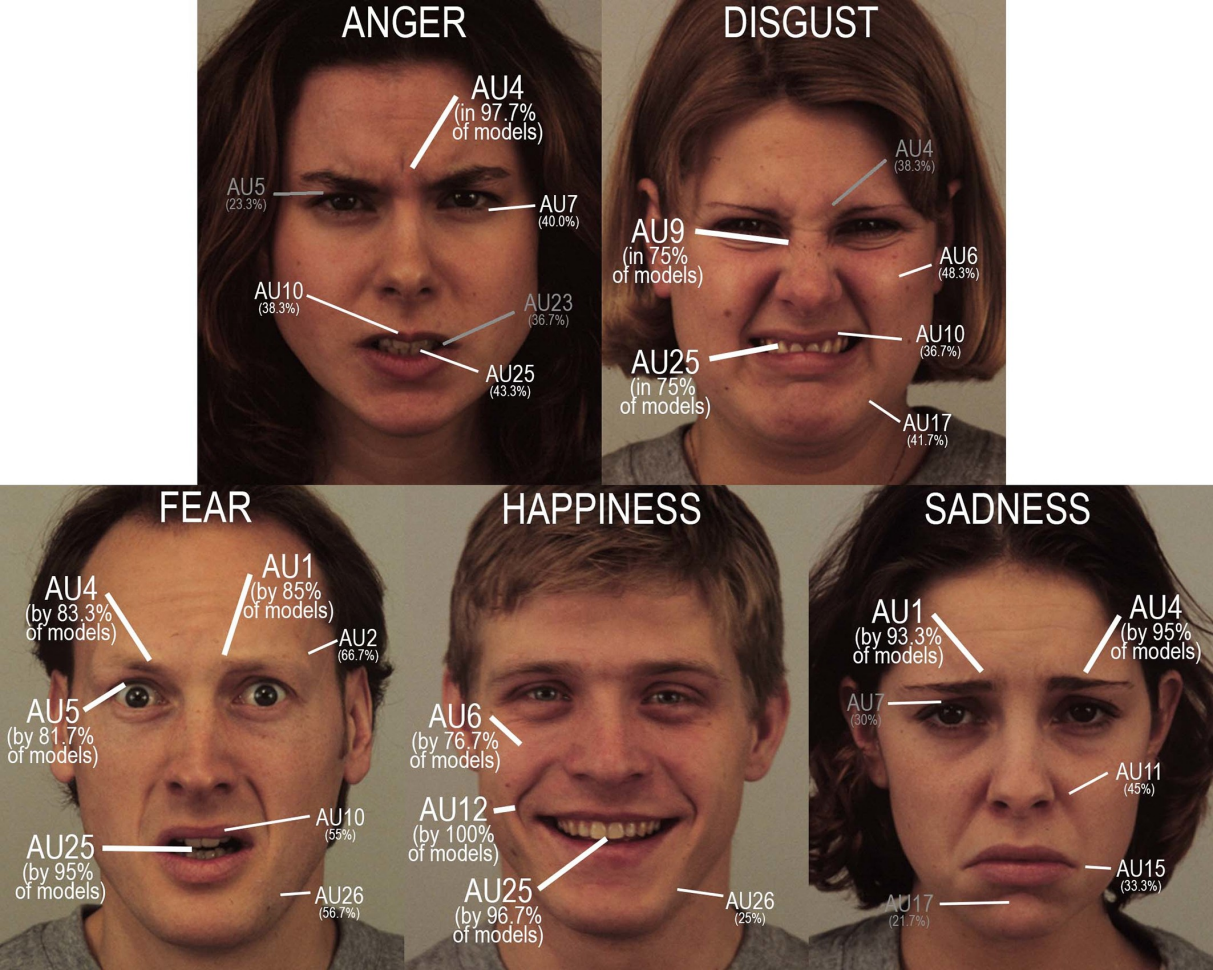
Illustration of the facial action units (AUs) that were used by the models to simulate the five emotions (with the example of the KDEF models, from the top to the bottom and from the left to the right: F29, F26, M10, M23 and F20). Their frequency is provided between brackets. **AU1**: inner brow raiser, **AU2**: outer brow raiser, **AU4**: brow lowerer, **AU5**: upper lid raiser, **AU6**: cheek raiser, **AU7**: lid tightener, **AU9**: nose wrinkler, **AU10**: upper lip raiser, **AU11**: nasolabial deepener, **AU12**: lip corner puller, **AU15**: lip corner depressor, **AU17**: chin raiser, **AU23**: lip tightener, **AU25**: lip part, **AU26**: jaw drop.

The relevance of a particular action for the accurate recognition of the emotion was investigated by computing the accuracy when this action was present, as well as the number of fixations and the latency to the end of last fixation for the accurate response, in both cases (with vs. without the facial action). We provide an illustration of facial AUs produced by the models for each emotional expression in Fig 1. Detailed results are provided in S1 Table.

The most frequent AU produced by the models to simulate *anger* was lowering the brow (AU4, used by 96.7% of the models). However, its absence did not hamper accurate recognition in the few models that did not use it. In those cases, anger was communicated by other actions, namely, parting the lips (AU25), tightening the lids (AU7), raising the upper lip (AU10), tightening the lips (AU23), and/or dropping the jaw (AU26). Furthermore, lowering the brow was never used alone, but always occurred with either one or several other facial actions, with the more frequent actions being parting the lips (AU25, used by 43.3% of the models), tightening the lids (AU7, 40%), raising the upper lip (AU10, 38.3%), tightening the lips (AU23, 36.7%), and raising the upper lid (AU5, 23.3%). The presence of only one of these actions gave rise to higher accuracy: lip tightening (AU23). Nose wrinkling (AU9), a typical action for disgust, was also used for anger by 8.3% of models, but with a detrimental effect on the number of fixations and time needed to make the correct response.

For the *disgust* expression, the two more frequent facial actions—wrinkling the nose (AU9) and parting the lips (AU25)—were used by 75% of models. Only the absence of nose wrinkling resulted in a significant decrease in accuracy, with significantly more fixations and a longer exploration time before the correct response was made. Two additional actions were produced by 48.3 and 41.7% of models: raising the cheeks (AU6) or the chin (AU17), respectively. However, only raising the chin allowed a significantly more accurate recognition, with fewer fixations and a shorter exploration time. Two actions were also used by more than a third of the models, despite their low reliability (i.e., their use led to a decrease in accuracy together with an increase in the number of fixations and time needed to make the correct response): lowering the brow (AU4, used by 38.3% of the models), and raising the upper lip (AU10, used by 36.7% of the models). These two actions are also part of the pattern used by the models to express anger, with brow lowering also being frequently used in fear and sadness expressions. Similarly, 13.3% of the models depressed the upper lip (AU16), and 8.3% raised their inner brows (AU1), resulting in lower accuracy, more fixations or longer latencies for correct responses. The first action was also used in a few occasions for anger, whereas the second action was the main one activated to express sadness.

The *fear* expression corresponded to the most complex pattern in the number of facial actions produced by the models: 7 AUs occurred in more than half of them. The most frequent AU was parting the lips (AU25, used by 95% of the models), but this action was also frequently present in other emotional expressions. Three other AUs were produced by more than 80% of the models, together with lips parting: raising the inner brows (AU1, 85%), lowering the brow (AU4, 83.3%), and raising the upper lid (AU5, 81.7%). Only the absence of the last action was associated with decreased accuracy despite more fixations and higher latencies, whereas brow lowering—a typical facial action in anger and sadness—triggered a decrease in accuracy for fear. Finally, the three other actions used by more than half of the models were raising the outer brows (AU2, 66.7%), dropping the jaw (AU26, 56.7%), and raising the upper lip (AU10, 55%), and none of these actions significantly increased accuracy. A few models also activated lip tightening (AU7, 5%), an action frequently used for anger, but it provoked a decrease in accuracy for fear.

All models performed the action of pulling the lip corners (AU12) to mimic *happiness*. Most models also parted their lips (AU25, used by 96.7% of models), and more than three-quarters raised their cheeks (AU6, 76.7%). Finally, 25% dropped their jaws (AU26). Given near maximal accuracy for recognizing happiness in nearly all models, no action emerged as being particularly beneficial or detrimental, except for raising the cheeks (AU6), which allowed the decoders to make the correct response after fewer fixations and a shorter latency. The presence of tightening lids (AU7) in mimicking happiness in 5% of models also resulted in the decoders making more fixations before making the correct response.

Finally, the *sadness* expression involved two main facial actions: lowering the brows (AU4, by 95%) and raising the inner brows (AU1, by 93.3%). Neither action was crucial since the accuracy was similar with or without them. Nevertheless, the correct response was made after fewer fixations and a shorter latency, and this gain was significant only when the inner brows were raised (AU1). Similarly, nasolabial deepening (AU11) and depressing the lip corners (AU15) were quite frequent (used by 45% and 33.3% of models, respectively), with no gain in accuracy but fewer fixations and faster responses when the lip corners were depressed. Approximately 30% of models also tightened their lids (AU7)—a typical action for anger—with a detrimental effect on accuracy and number of fixations/length of latency before a correct response. Two less frequent actions also had a detrimental effect on accuracy: parting the lips (AU25, 8.3%) and raising the upper lip (AU10, 6.7%).

#### Exploratory pattern for correct recognition

**Fig 2:**
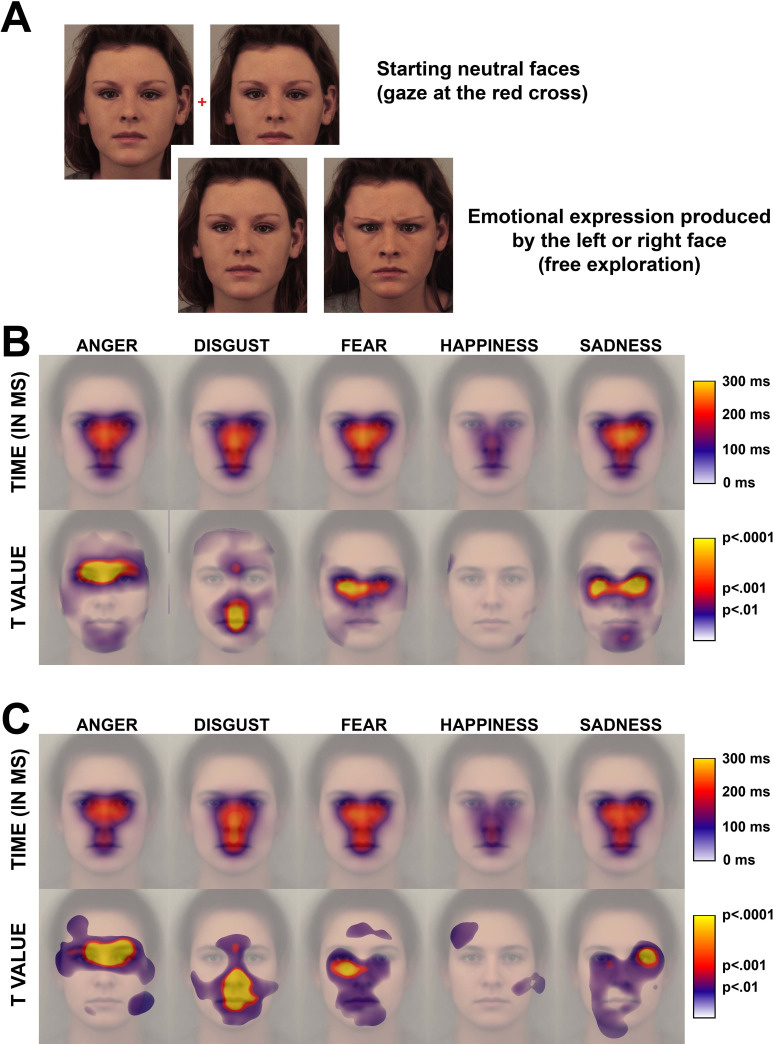
A) Illustration of the procedure. Each trial started with the neutral face of a model displayed on each side of a red cross (with the example of the KDEF model F09). Participants were instructed to look at the red cross. After a 1-sec fixation, one of the two faces was replaced by an expressive face, simulating the occurrence of an expression. B) Mean time and T values for the 5-choice task (illustrated on the prototypical face). For Time, the color code of a pixel indicates the average time the pixel was in the participants’ foveal zone. For T values, the color code indicates the *p* value after a Student’s T-test that compared the mean time for the emotion to the mean time for the four other emotions. Only increased time is reported. C) Mean time and T values for the Yes/No task, with the same rationale as in B.

To directly compare the facial areas that were explored by the decoders for the different facial expressions, we transformed the gaze behaviors to fit them to the same prototypical neutral face (see Method section). The visual exploratory patterns for correct recognition are illustrated in Fig 2B for the 5-choice task and in Fig 2C for the Yes/No task. The first row displays the mean time the participants gazed at the different facial areas, according to the facial emotions the models mimicked. The color code for the pixels corresponds to the approximate time the facial region was within the foveal zone. The second row contains the maps of Student’s T-test values (and corresponding *p* values), indicating the facial areas that received significantly longer gazes, that is, visual attention, for a particular facial emotion than for the other facial emotions. The figures showing mean time by area indicate a similar pattern for all facial emotions in both tasks, with longer time spent gazing in the central face region. This reflects the fact that the participants’ first fixations were devoted to centralizing the main facial features before exploring them [25, 26]. The participants gazed at nearly all the internal features for the negative emotions, whereas for happiness, the fixations were mainly directed to the center of the face (the tip of the nose) and the mouth; for this emotion, the participants rapidly reached the correct answer after few fixations. For negative emotions, the nose region that received more gazes varied slightly across emotions, and this is more evident in the Yes/No task, with more time at the top part of the nose for anger and more time at the middle part for disgust. T-tests further showed that the participants gazed more at regions where relevant facial actions were present in both tasks. For anger, they gazed more at the regions of eyebrows, where the brows were lowered by most models (AU4). For disgust, they gazed more at the nose, which was wrinkled (AU9), and at the mouth, where the lips were parted (AU25), the upper lip was raised (AU10), and the chin was dropped (AU17). The region at the top of the nose and between eyebrows also received more gazes for disgust. This may have corresponded to gazes at the lowered eyebrows or morphological modifications due to nose wrinkling that extended to this region. For both fear and sadness, the facial area that received more visual attention was the region of the eyes, excluding eyebrows, in agreement with the use of several facial actions in this region for both facial emotions (see Fig 1). This additional attention was mainly devoted to the right eye for fear and more bilateral/left-sided for sadness. The similarity of the pattern in the two tasks/experiments further suggests that the exploratory patterns were quite the same whether the exploration was mainly stimuli-driven (in the 5-choice task) or expectation-driven (in the Yes/No task).

#### Exploratory pattern that sustained misidentifications of facial emotions

**Fig 3:**
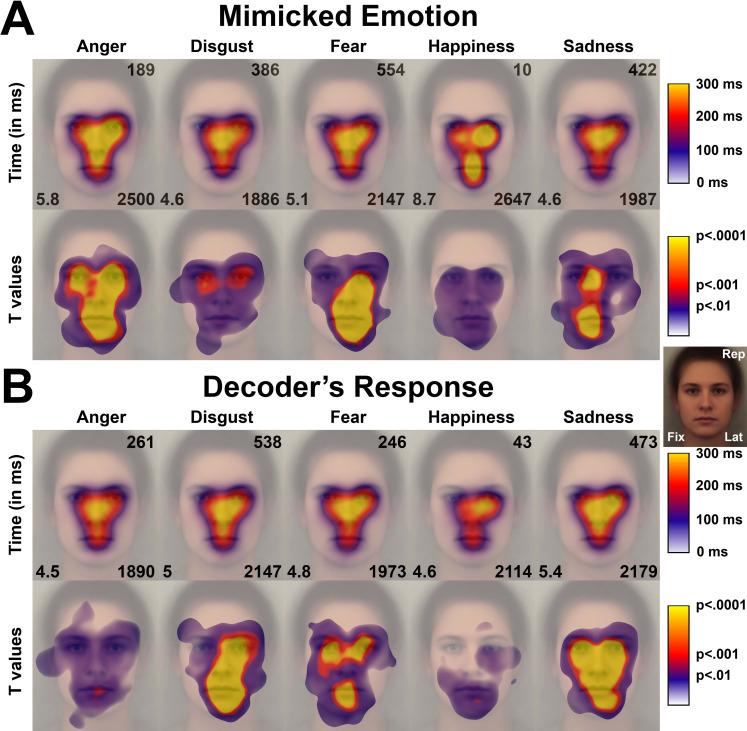
Exploratory patterns for false recognition in the 5-choice task (illustrated on the prototypical face). A) Mean time (first row) and T values (second row) when the participants perceived an emotion other than the mimicked one (e.g., perceived disgust, fear, happiness, or sadness when the models mimicked anger). B) Mean time (first row) and T values (second row) when the participants perceived an emotion in other mimicked emotions (e.g., perceived anger when the models mimicked disgust, fear, happiness, or sadness). The values at the corners of faces indicate the number of such errors in the course of the experiment (top right, *Rep*), the number of fixations (bottom left, *Fix*) and the latency from stimulus display to the end of the last fixation to a response (bottom right, *Lat*), as illustrated in the small face on the right.

Next, we investigated the exploratory pattern for errors, i.e., when the decoders did not recognize the facial emotions mimicked by the models. Two non-exclusive sources of errors were considered. First, the decoders may have missed the significant signals. In that case, the decoders did not recognize the mimicked emotion because they did not gaze enough to the relevant facial action (e.g., eyebrows lowering in anger). Second, the decoders may have focused their visual attention on irrelevant facial actions. In that case, the irrelevant action would have oriented the decoders’ response toward a particular facial emotion (e.g., responding “anger” after having gazed at the eyebrows in a disgusted face). To investigate these issues, we computed two types of exploratory maps (see Fig 3A and 3B). The first type (Fig 3A) illustrates the pattern of gazes applied to a given mimicked facial emotion when the decoders did not recognize it; for example, the exploratory pattern for angry models when the decoders perceived another emotion. This figure clearly shows that the decoders did not miss the relevant facial actions. In contrast, in most cases, the decoders made more fixations and gazed longer at the face when they failed to recognize the mimicked emotion than when they succeeded. These fixations and gazes included the relevant regions, for example, the eyebrows in angry faces, the wrinkled nose in disgusted faces, or the eyes in fearful and sad faces. This conclusion was reinforced by applying Student’s T-tests between the patterns for errors and the patterns for correct answers (illustrated in Fig 2) (see Fig 3A, second row). This analysis showed that the differences were positive (i.e., indicating longer times for errors) for nearly all facial regions, including the regions of the relevant facial actions. Furthermore, many regions that did not support relevant facial actions for the actual emotion, received significantly more gazes when the decoders failed to recognize the mimicked emotion than when they succeeded. This was the case for the middle and lower facial regions in anger, the eyes in disgust, and the nose and mouth regions in fear and sadness.

The second type of exploratory patterns (Fig 3B) shows the regions that were gazed at when the decoders (falsely) recognized each kind of emotion (e.g., where they gazed at when they perceived anger in another facial emotion). These visual patterns further show that the decoders’ responses were associated with enhanced exploration of regions that supported facial actions for the emotion that was falsely perceived, suggesting that the decoders perceived elements in these regions that reinforced the error. That is, the decoders who falsely perceived disgust, fear, or sadness gazed more to the regions that sustained correct recognition (see Fig 2), namely, the nose for disgust and the eyes for fear and sadness. Nevertheless, the decoders also gazed at other regions, e.g., the mouth region in most cases. Remarkably for anger, the mouth region was the main region associated with the misperception of this emotion. The models frequently used three facial actions at the level of the lips to mimic anger: raising the upper lip (AU10), tightening the lips (AU23) and parting the lips (AU25). These actions were also present in other facial emotions; lip raising was frequent in disgust and fear (and, to a lesser extent, in sadness; see S1 Table), and lip parting was frequent in all facial emotions. For both disgust and fear, the additional exploration of the mouth also indicated the search for informative facial action(s) in this region. However, both disgust and fear involved the same relevant AUs, that is, actions also relevant for anger expression (upper lip raising, AU10, and lip parting, AU25). Thus, this strategy increased the probability of associating such actions with a facial emotion other than the mimicked one. The same rationale applies to sadness and happiness. Interestingly, happiness was the only facial emotion for which misperception was associated with a lower exploration of some facial regions, namely, the top part of the face. In those cases, the decoders falsely perceived happiness in other expressions after having both tended to over gaze at the mouth regions but also ignored the rest of the face, where they would have found facial actions indicating other (negative) facial emotions. However, caution is warranted in interpreting these results, given the extremely low number of available trials.

Thus, errors were mainly driven by the additional exploration of facial regions with less relevant or irrelevant facial actions, orienting the participants’ responses to an incorrect emotional expression. We further investigated whether the kinds of errors also varied according to the exploratory pattern that was applied; in other words, whether the participants who did not recognize a given emotional expression (e.g., anger) exhibited a distinct pattern related to the emotion they falsely perceived (i.e., disgust, fear, happiness, or sadness). These patterns are illustrated in S5 Fig. Overall, despite a global tendency to gaze more at the mouth region (as already highlighted previously), these patterns support the hypothesis that when the participants missed an emotion, they tended to over gaze at particular regions related to the emotion they perceived rather than the actual displayed emotion. More specifically, the regions they gazed at were regions that (supposedly) supported facial actions for the emotion they perceived. For example, the participants who saw disgust in an angry face over gazed at the nose area, those who saw fear over gazed at the eyes region, and those who saw sadness over gazed at a region between the nose and the left eye; all these areas were relevant for the accurate recognition of these emotions, as illustrated in Fig 2. In the same way, the participants who perceived disgust in a sad face over gazed at the nose region, a pattern not evidenced for the other kinds of errors.

## Experiment 2

In the previous experiments, we investigated the relation between the production of facial configurations and visual exploratory patterns in both accurate and false recognition of emotional expressions. Here, we investigated whether the distinct facial actions produced by the models were necessary and/or sufficient to accurately recognize facial emotions. To do that, we asked two new groups of participants to recognize a subset of the previously used facial expressions. For one group, we investigated the necessity of facial actions by displaying them with expressions in which we removed the facial actions occurring at the level of one of three facial areas (either the eyes and eyebrows, the nose and cheekbones, or the mouth and chin; group “without the area”). For the second group, we investigated whether these different sets of facial actions were sufficient to recognize facial emotions by displaying facial actions at one of the three previous facial areas alone (group “with only the area”). In both groups, the accuracy with the partial emotional expressions was compared with the accuracy when all facial actions were present. Gaze behaviors were used to manage the start of trials, but were not analyzed in the current experiment.

### Methods

#### Participants

Sixty-four new individuals were included and separated into two groups with 32 participants in each: the group “without the area” [16 females, mean age = 22.0 ± 2.0 (SD) years, range 20.0–28.0 years; 16 males, mean age = 24.1 ± 4.0 (SD) years, range 18.0–36.0], and the group “with only the area” [16 females, mean age = 21.4 ± 1.7 (SD) years, range 19.0–26.0 years; 16 males, mean age = 23.3 ± 2.5 (SD) years, range 20.0–28.0]. Three additional participants were excluded due to calibration difficulties. All participants reported normal or corrected-to-normal vision, and no participant reported a history of psychiatric or neurological disorders. The participants provided written informed consent prior to the experiment and received financial compensation. Testing was conducted in accordance with the Declaration of Helsinki and approved by the French ethics committee (CPP Sud-Est III—2016-A02056-45).

#### Materials

We used a subset of 16 models from Experiments 1a and 1b (8 females: F05, F10, F12, F19-F21, F27, and F32; 8 males: M13-M14, M16, M23-M24, M31-M32, and M34). These models were selected because at least 75% of participants correctly recognized each mimicked emotion in the 5-choice task (anger: mean = 95.6% correct, range = 85–100; disgust: mean = 93.75% correct, range = 80–100; fear: mean = 90.9% correct, range = 80–100; happiness: mean = 100% correct, range = 100–100; sadness: mean = 90.9% correct, range = 75–97.5). Two other models (F09 and M11) were used for training sessions. To create the partial expressions, three parts were isolated in the original expressive face and placed on the neutral face of the same person (see S2 Fig). The *top part* included the forehead, the eyebrows and the eyes. It was delimited by the hairline, borders of the face, and bottom limits of the eyes, including eye sockets. The area between the eyebrows, except for the top part of the nose, was also included. The *middle part* included the nose and cheekbones. The borders were below the eye sockets and the area between the eyebrows, face borders, and bottom limits of cheekbones. The *bottom part* included the mouth, jaws and chin; the border being delimitated by the face borders on each side and at the bottom, and the cheekbones and nose at the top. Two types of partial facial expressions were created: (i) one composed of actions from two parts of the face that were superposed on a neutral face, thus creating emotional expressions without the third part of the face, and (ii) the other was a single part superposed on a neutral face, creating a partial emotion with only one expressive region.

#### Procedure

Following the explanations about the purpose of the study, the precise task they will have to perform and signature of consent forms, the participants sat in front of the monitor and were instructed to put their head on a chin-rest at approximately 60 cm from the screen. A thirteen-point calibration was performed. The emotion recognition phase was performed after the calibration was successful (i.e., the target ideal calibration was a less than 1.5° lag in the x and y dimensions). During the recognition phase, each trial started with two neutral faces of the same model on each side of a red cross, which was displayed at the center of the screen. The participants were told to gaze at the red cross for 1 sec to trigger an emotional expression. After 1 sec, the left or right neutral face was replaced by an expressive face of the same model.

The task was the same as in Experiment 1a (i.e., the 5-choice task), except that the expression displayed was presented either fully expressed, or partially expressed. For half of the participants, the partial expressivity was tested with only one expressive area (bottom part, top part, middle part), the rest of the face being neutral. For the other half, the partial expressivity was tested with one unexpressive area (bottom part, top part, middle part), the two other areas expressing the same emotion. This time, participants had to perform 320 trials: 16 identities (8 females/8 males) x 5 emotional expressions x 4 versions (the expression expressed over the full face, bottom part, top part, or middle part). The trials were parted in 4 blocks of 80 trials each. I.e., in 1 block: each of the 16 identities was presented with the 5 expressions, and each expression was presented either in the full face, bottom part, top part, or middle part condition. Each identity was thus presented twice in one of the 4 conditions in each block but for two different emotions. As in previous experiments, the frequencies of the different models, expressions, versions, and sides were equalized across blocks, and the four versions of a picture were displayed in separate blocks. The order of blocks and left/right positions was alternated between participants. Participants trained with 40 trials including 2 identities (1 female/1 male) x 5 emotional expressions x 4 versions.

#### Data analyses

In the two partial emotion recognition tasks, we tested for the effect of removing or displaying each facial region alone by computing two types of Student’s T-tests to investigate whether (i) the accuracy significantly differed from the fully expressed emotion and (ii) the accuracy was significantly above chance. Thus, the first type of T-test contrasted the accuracy in each partial emotion condition with the accuracy in the full emotion condition. The second contrasted the accuracy in the partial emotion condition to chance level (i.e., 20%). Bonferroni corrections for multiple comparisons were performed in both cases, with 15 comparisons in each group (3 partial emotions x 5 emotions).

### Results

**Fig 4:**
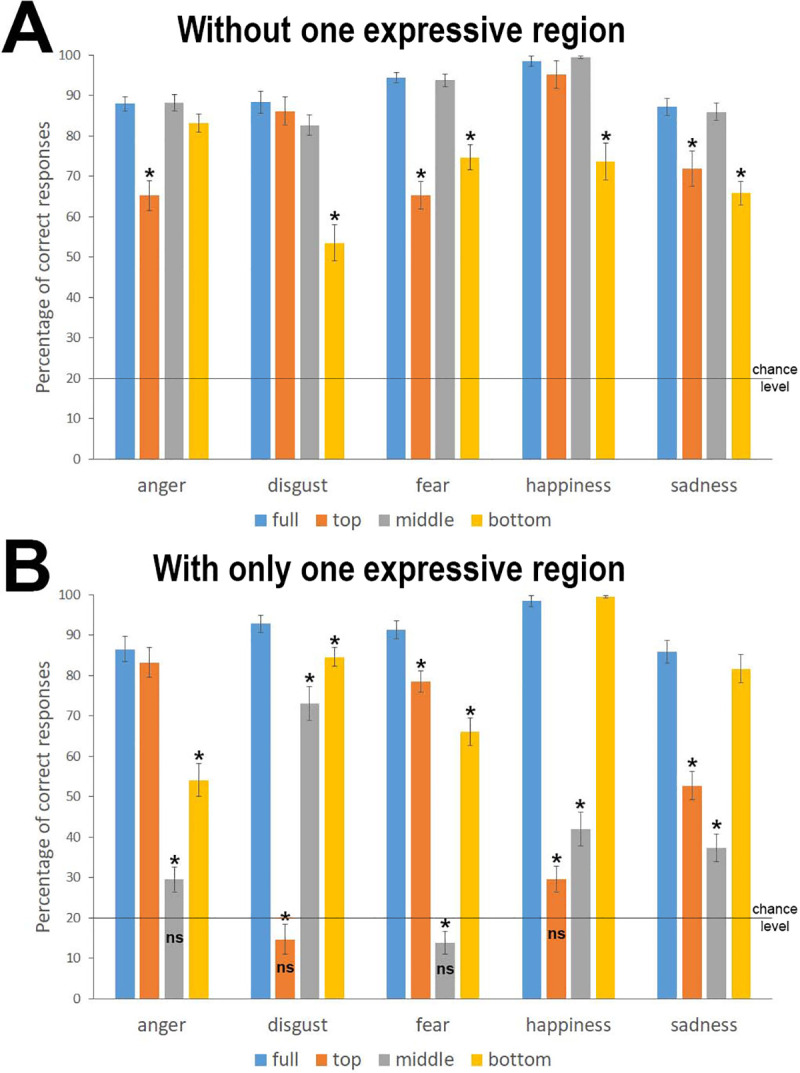
Percentage of correct recognition for the different emotion categories. A) In the partial emotion recognition task. B) When displayed alone. Errors bars are SE. *: significantly different from the full expression; ns: non-significantly different from chance (i.e., 20%). All comparisons were performed using Student’s T-tests, with Bonferroni corrections for multiple comparisons.

We summarize the mean percentages of correct recognition for each emotion based on these manipulations in Fig 4. The distribution of responses in the different emotion categories for the different conditions is available in S6 Fig.

Fig 4A indicates that, overall and despite variations in performance, the accuracy remained well above chance (i.e., 20%) when any expressive region was removed, regardless of the expression. This indicates that the emotional cues were communicated by a complex set of facial actions and that no single AU was necessary. Nevertheless, some facial actions are sufficient since the accuracy was at the same level as in the full expression when these actions were isolated (Fig 4B). Overall, AUs in the middle part of the face were of little effectiveness: emotional expressions were well recognized without them, and the accuracy was very low when they were the only ones available. The pattern was more variable across emotions for the facial actions in the top or the bottom regions.

For *anger expression*, the facial actions in the region of the eyes and eyebrows appeared important, if not crucial: only their removal strongly reduced accuracy, and accuracy was similar to the full expression when only facial actions in this region were displayed. The information in the mouth region alone may support the recognition of anger but with a low accuracy (<60%); in this case, the participants frequently perceived sadness in the face (see S6A Fig). Interestingly, anger was the only expression with an accuracy for full faces that was lower than in the 5-choice task. It seems that by adding expressive features to the neutral face, we removed an important signal for this emotion: moving the forehead forward. The recognition of *disgust* was mainly impaired by the absence of actions in the bottom part of the face; in those cases, the participants frequently saw anger. Alone, this region allowed good recognition (>80%). The nose alone supported quite good accuracy (>70%) and was the only exception regarding the low expressiveness of this region. The disgust expression in the eye region alone provoked the perception of anger in most cases (>75%, see S6A Fig). The *fear* expression displayed a more complex visual pattern in relation to the variety of facial actions used by the models: the accuracy was non-drastically but significantly decreased by removal of actions from either the top or the bottom of the face, and these two regions alone allowed a good accuracy, although accuracy was below that of the full expression. There was a slight advantage regarding information from the eyes’ region, nevertheless. Both the removal of facial actions at the eyes or the display of facial actions in the mouth region alone favored the misperception of disgust (see S6A and S6B Fig). The removal of facial actions at the mouth regions or the display of facial actions at the eyes’ region alone favored the misperception of sadness. Unsurprisingly, *happiness* was mainly conveyed by the mouth: only its absence significantly impaired the recognition of happiness, and accuracy was at 100% with only this source of information. Nevertheless, the eyes and nose regions also played a non-silent role since they supported good accuracy when they were both present (>70% when the mouth was removed, i.e., only the eyes and nose regions were displayed; between 30 and 45%, when the eyes or the nose regions were the only expressive regions, respectively). *Sadness* followed a similar pattern as fear—the recognition was supplied by both the facial actions in the top and the bottom regions, with neither being as informative as the full expression; however, there was a slight advantage for the actions in the bottom region of the face. The main misperception when the eyes region was displayed alone was fear, whereas it was disgust when the mouth region was alone.

These results also indicated that the accuracy in communicating the whole set of facial emotions is not the same for the different facial regions. The middle region was quite effective only for signaling disgust. The top region of the face is reliable for anger expression but also, to a lesser extent, for fear expression. It may be reliable for sadness, but not very good (approximately 50%). The participants were at chance level when only the top region of the disgust and happiness expressions were shown. In contrast, the bottom part of the face was quite reliable for all emotion categories: the bottom part supported an accurate recognition that was above 50% and 60% for anger and fear, respectively, above 80% for disgust, and at nearly the same level as the full facial expression for sadness and happiness. Thus, the facial actions in the bottom part of the faces appeared to be more effective in efficiently discriminating all the facial emotions manipulated in the course of this study.

## Discussion

Several conclusions can be drawn from the present study. The first conclusion was that the facial expression of basic emotions encompassed a complex pattern of facial actions. The emotional expressions that were recognized with high accuracy from few actions (namely, happiness and anger) benefited from additional actions that allowed faster and/or more accurate recognition. This benefit held for happiness, which was recognized faster when actions in the middle and top parts complemented the actions displayed at the mouth level. This benefit was more apparent for anger, which was never exclusively communicated by lowering the eyebrows but was always accompanied by complementary actions in other parts of the face, including the bottom part. Indeed, lip tightening (AU23) gave rise to higher accuracy. This may be explained by the fact that lowering the brow is an action that is also involved in other emotional expressions. Thus, information at the mouth level can be used by observers to confirm their first hypothesis on the expression displayed. This may be useful for example, to discriminate anger from disgust (which is frequently confounded with anger) thanks to AUs displayed in the mouth region. The three other emotions (disgust fear, and sadness) recruited a larger set of facial actions. The participants gazed more at the nose, which was wrinkled (AU9) in processing disgust, and at the mouth, where the lips were parted (AU25), the upper lip was raised (AU10), and the chin was dropped (AU17). They allocated more visual attention to the region of the eyes for both fear and sadness. These results are partly consistent with those of the literature, which reports that the mouth is important to process disgust but not solely [e.g., 31], that the recognition of fear could be based on the eyes/eyebrows [e.g., 32, 36–39] but also on the mouth [31], and that sadness recognition could depend on both eyes/eyebrows and mouth areas [32, 36–39]. Nevertheless, in our study, no individual action supported the highest level of accuracy in communicating emotional states; on the contrary, many of them were of low reliability. At the same time, no single facial action was necessary to communicate an emotional state, with accuracy far above chance regardless of the lack of facial actions. Finally, the coding of facial actions revealed that many actions were frequently mobilized for several emotions, which renders a strategy that would associate a single action to a single emotion category quite inefficient. In contrast, the facial communication of the full panel of emotional states relies on a complex configuration of entangled facial actions.

In this framework, the second conclusion was that misunderstanding facial emotions rarely resulted from missing the relevant facial actions: the participants gazed at the relevant regions enough to recognize the emotion that the models attempted to mimic. However, the information they gathered was not clear enough to recognize the emotion, and the participants searched for that additional information in other facial regions. The only exception in our study was the false perception of happiness in another emotion, which was associated with a lack of exploration of features other than the mouth; however, this kind of error was very uncommon. In most cases, false recognition came after the decoders extended their explorations to other, less relevant facial actions. Then, their decisions were turned toward another emotion, either because they gazed at actions that were ambiguous (i.e., relevant for several emotions such as, for example, raising the upper lip as used for anger, disgust and fear) or because their visual attention was attracted by a region that usually sustained actions relevant for other emotions (for example, gazing at the nose in angry faces, thereby orientating the response toward disgust). The similar patterns in the two tasks further suggest close exploratory patterns whether the exploration was mainly stimuli-driven (in the 5-choice task) or expectation-driven (in the Yes/No task).

It is noteworthy that the confusions essentially took place between negative emotions; namely, anger, disgust, fear, and sadness. Happiness was characterized by faster (i.e., after fewer fixations) and more reliable (with almost no errors) recognition, with fewer false recognitions in another expression. This may reflect a particular status of the discrete category of happiness. However, it should also be noted that it was the only positive expression in our design, what may also explain our results. In this regard, Leleu et al. (2018) observed 3 components in the cerebral response to facial expressions, corresponding to different stages of their processing; evidence of a categorical response was found only for the third and last one [49]. Our results could thus reflect the final stage of expression recognition, where the visual exploration of the different facial actions would aim at distinguishing close expressions, for example because of their identical valence. This explanation remains tentative, however, and requires further investigation, such as investigating several positive expressions, and/or balancing the expressions according to their hedonic valence.

A final remarkable conclusion was the particular status of the bottom facial region. This region was not gazed at to the same extent as the central and top regions when the participants reached the correct response, especially for negative emotions. Additionally, among the relevant facial actions that attracted more visual attention for the different emotions, most were in the top or middle regions, as illustrated in Fig 2 [see also 35]. This is consistent with research that shows a particular focus on the eye area when recognizing expressions [50] or, in general, the importance of eye contact in nonverbal communication and social interaction [51, 52]. At the same time, the additional looking time reported for errors was frequently directed toward the lower part of the face, as evidenced in Fig 3. Thus, this revealed a relation between the additional focus to the lower facial region and increased uncertainty about the actual facial emotion that was mimicked. In other words, participants turned their gaze toward the lower part of the face when they could not determine the expression after focusing on the upper part. Moreover, our results on partial emotional expressions also revealed that the bottom part was the only facial region that allowed, from itself, to discriminate all facial emotions from each other with an above-chance accuracy. In other words, looking at the mouth region is the most efficient strategy for those who have difficulties integrating the complex information from the top part—which includes several subtle actions at the level of both the eyes and eyebrows—or integrating information of actions from distant parts of the face at once. In that way, many studies reported that neurological or psychiatric patients with impaired facial emotion recognition skills exhibit more attention to the mouth [e.g., [13, 14, 53, 54]]. This behavior might thus reflect an (effective) adaptation to their actual difficulties in recognizing facial emotion by means of the usually most appropriate exploratory strategy, i.e. by looking at the upper part of the face.

It is noteworthy that, contrary to the literature, our results do not indicate any particular asymmetry in the hemi-face used to recognize the different expressions [55–60]. This can result from the procedure we used, displaying the expressive face to the left or to the right of the fixation cross (the participant being instructed to focus her or his gaze on this cross before we present the expression). With such a procedure, subjects tended to first explore the hemi-face closest to their initial fixation (for a similar observation, see also [57]). The preference for the right or left hemi-face was thus driven more by this spatial proximity than by the role of each hemi-face in the production and recognition of expression. Since the expressive faces were equally displayed on the left and right side of the fixation cross, the fixations were almost equally distributed between the two hemi-faces, thus hiding the potential asymmetry of the two hemi-faces.

In conclusion, the present study stressed that recognizing emotions from facial expressions is a complex process that involves the integration of various sources of facial cues. Furthermore, successful decoding of facial emotions involves both actively exploring relevant facial actions and disentangling these relevant cues from less relevant and confusing ones. In that framework, mistaking one emotion for another frequently comes after difficulty in distinguishing emotions based on supposedly relevant actions. This forces the decoder to search for additional information in less relevant regions. Here, the complex entanglement of relevant actions between emotions leads the decoders either to make a decision from facial cues that are shared by several emotions and are thus more ambiguous or to orient their perception from a cue that is not relevant for the emotion the emitter is currently expressing.

## Supporting information

S1 Fig Illustration of gaze transformation. The first column illustrates the fixation locations for a participant when exposed to the picture (with the example of the KDEF model F09). In the second column, a cumulative density function for normal distributions with an asymptote at the fixation location that corresponded to the fixation duration was applied. The mean was 96 pixels around (approximately 1 degree of visual angle, i.e., the approximate size of the foveal zone). By adding the duration for each pixel, we obtained exploratory maps applied to the picture, with color codes indicating the approximate time each pixel/area was within the foveal zone. In the third and fourth columns, the coordinates of fixations were transformed to match a single neutral prototypical face (third column), and corresponding transformed exploratory maps were computed (fourth column). By averaging pixel by pixel the exploratory maps for the different participants and pictures, we obtained pictures that are contained in the manuscript and applied statistics to them.
(TIF)

S2 Fig Illustration of the partial expressions used in the study (with the example of the KDEF model F12). For each expression (top left), the top, middle and bottom parts were isolated (top right) and superimposed on the neutral expression of the same person to obtain a full expression (bottom left). Partial expressions were created either by removing one part from the full expression (bottom right, first row) or by superimposing only one part on the neutral face (bottom right, second row).
(ZIP)

S3 Fig Percentage of correct responses, number of fixations and latencies to the end of the last fixation to a correct response during the 5-choice (N = 40) and the Yes/No (N = 40) tasks, according to facial emotions and gender of the models. Error bars are SE.
(TIF)

S4 Fig Repartition of responses during the 5-choice recognition task. A) Percentage of time each emotion was perceived in a model that expressed another emotion, according to the emotion that was perceived and the gender of the model (e.g., for anger, percentage of time the decoders responded “anger” when the models simulated either disgust, fear, happiness, or sadness). B) Repartition of the responses (in %) for the five emotions expressed by the models, according to the response and model’s gender. Error bars are SE.
(TIF)

S5 Fig Exploratory patterns for false recognition in the 5-choice task (illustrated on the prototypical face). Mean time (large face) and T values (small face) according to the emotion the model attempted to mimic and the response of the decoder. The values at the corners of the faces indicate the number of such errors over the course of the experiment (top right), the number of fixations (bottom left) and the latency to the end of last fixation for a correct response (bottom right). na: not applicable.
(TIF)

S6 Fig Repartition of responses during the partial expression recognition task. A) The percentage of time each emotion was perceived according to the emotion and the facial region that was removed. B) The percentage of time each emotion was perceived, according to the emotion and the facial region that was displayed alone. Error bars are SE. For the statistical comparison of correct responses in the different conditions, see Fig 4 in the manuscript.
(ZIP)

S1 Table (ZIP)
