# Supplementary figures and images for: The spatial distribution of eye movements predicts the (false) recognition of emotional facial expressions
Source: PLoS One. 2021 Jan 26;16(1):e0245777. doi: 10.1371/journal.pone.0245777 (PMC7837501; doi:10.1371/journal.pone.0245777)

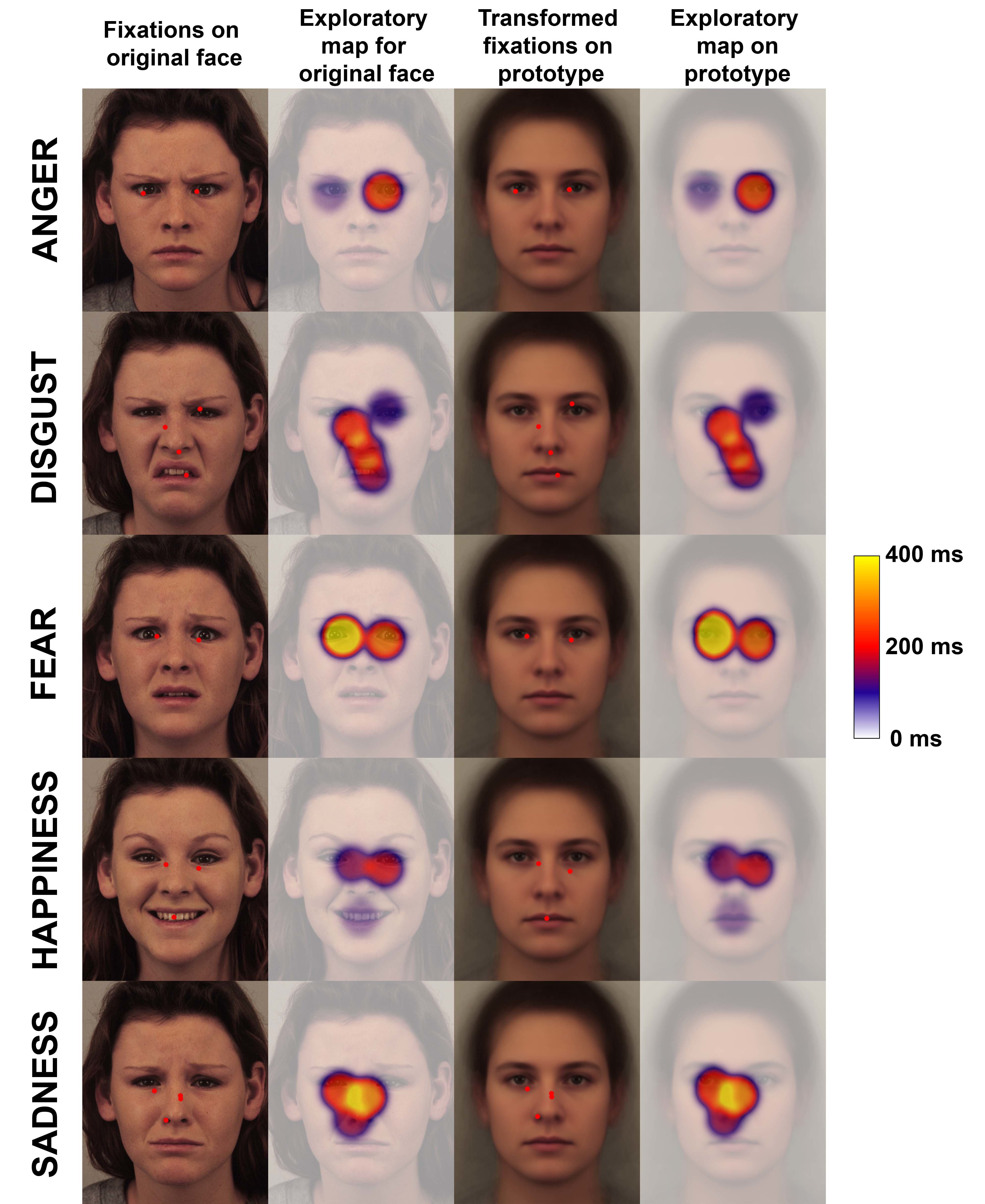

Supplement: S1 Fig — The first column illustrates the fixation locations for a participant when exposed to the picture (with the example of the KDEF model F09). In the second column, a cumulative density function for normal distributions with an asymptote at the fixation location that corresponded to the fixation duration was applied. The mean was 96 pixels around (approximately 1 degree of visual angle, i.e., the approximate size of the foveal zone). By adding the duration for each pixel, we obtained exploratory maps applied to the picture, with color codes indicating the approximate time each pixel/area was within the foveal zone. In the third and fourth columns, the coordinates of fixations were transformed to match a single neutral prototypical face (third column), and corresponding transformed exploratory maps were computed (fourth column). By averaging pixel by pixel the exploratory maps for the different participants and pictures, we obtained pictures that are contained in the manuscript and applied statistics to them. (TIF) [file pone.0245777.s001.tif]

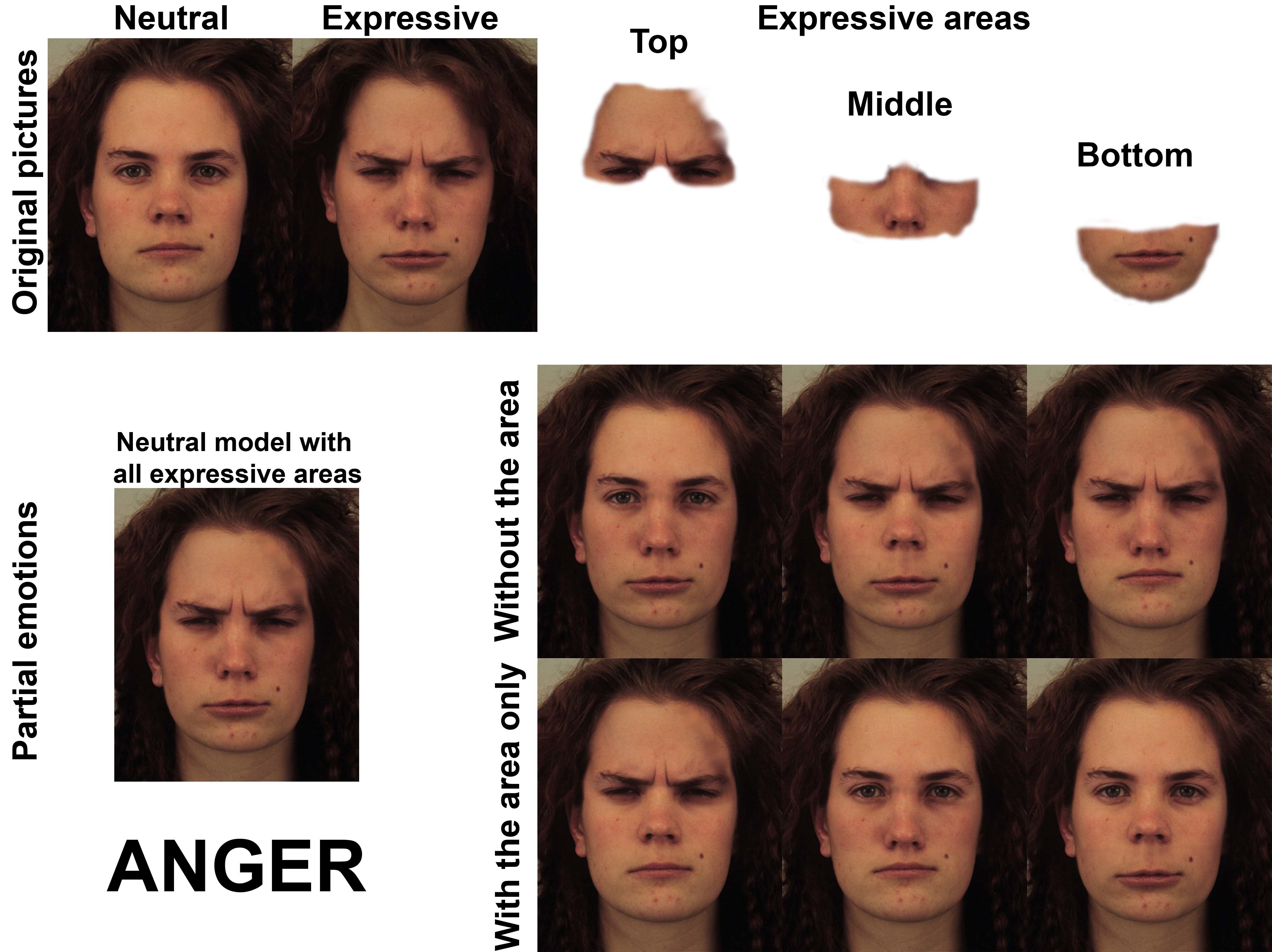

Supplement: S2 Fig — For each expression (top left), the top, middle and bottom parts were isolated (top right) and superimposed on the neutral expression of the same person to obtain a full expression (bottom left). Partial expressions were created either by removing one part from the full expression (bottom right, first row) or by superimposing only one part on the neutral face (bottom right, second row). (ZIP) [file pone.0245777.s002.zip › S2_Fig (anger partial).tif]

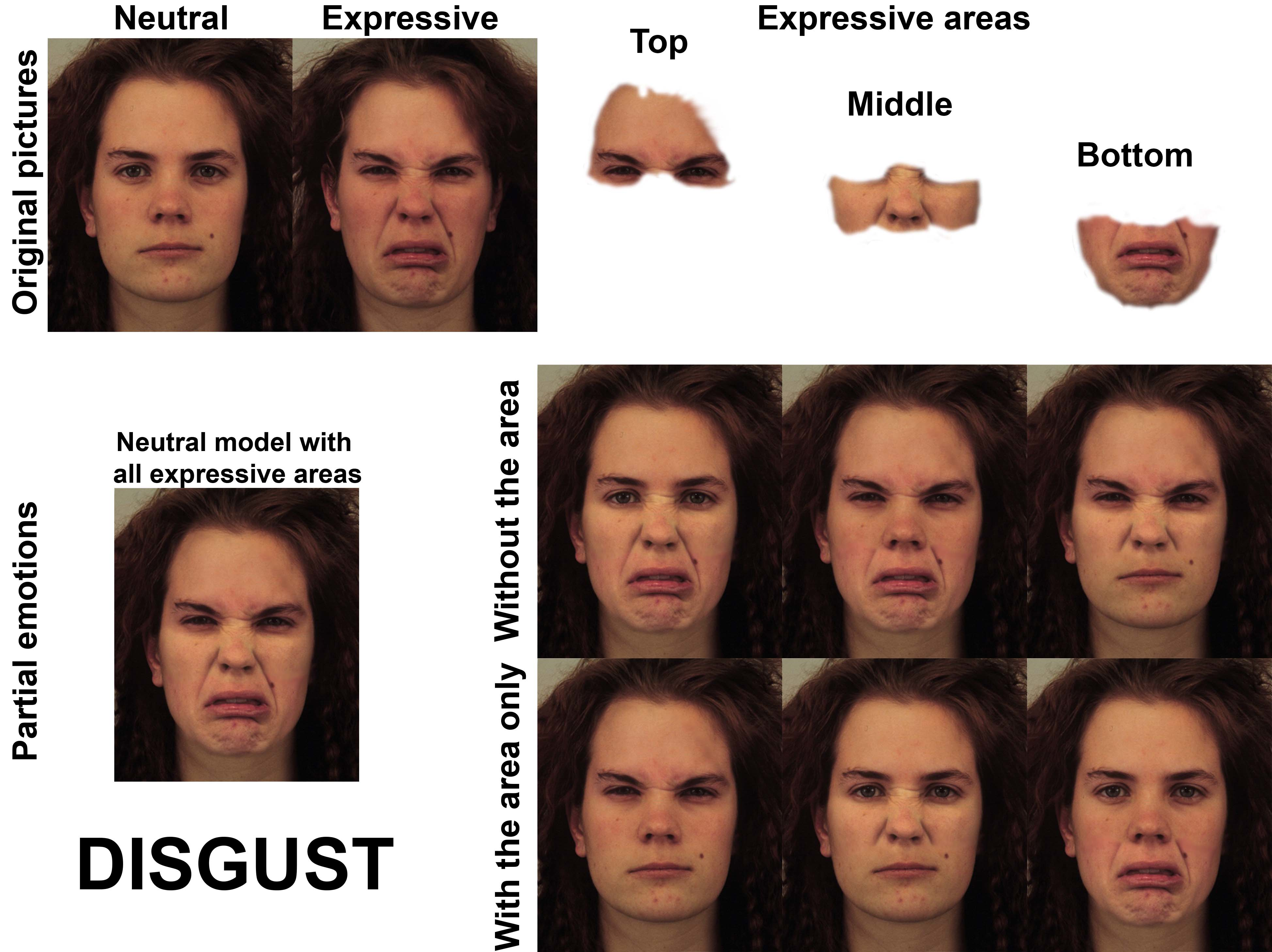

Supplement: S2 Fig — For each expression (top left), the top, middle and bottom parts were isolated (top right) and superimposed on the neutral expression of the same person to obtain a full expression (bottom left). Partial expressions were created either by removing one part from the full expression (bottom right, first row) or by superimposing only one part on the neutral face (bottom right, second row). (ZIP) [file pone.0245777.s002.zip › S2_Fig (disgust partial).tif]

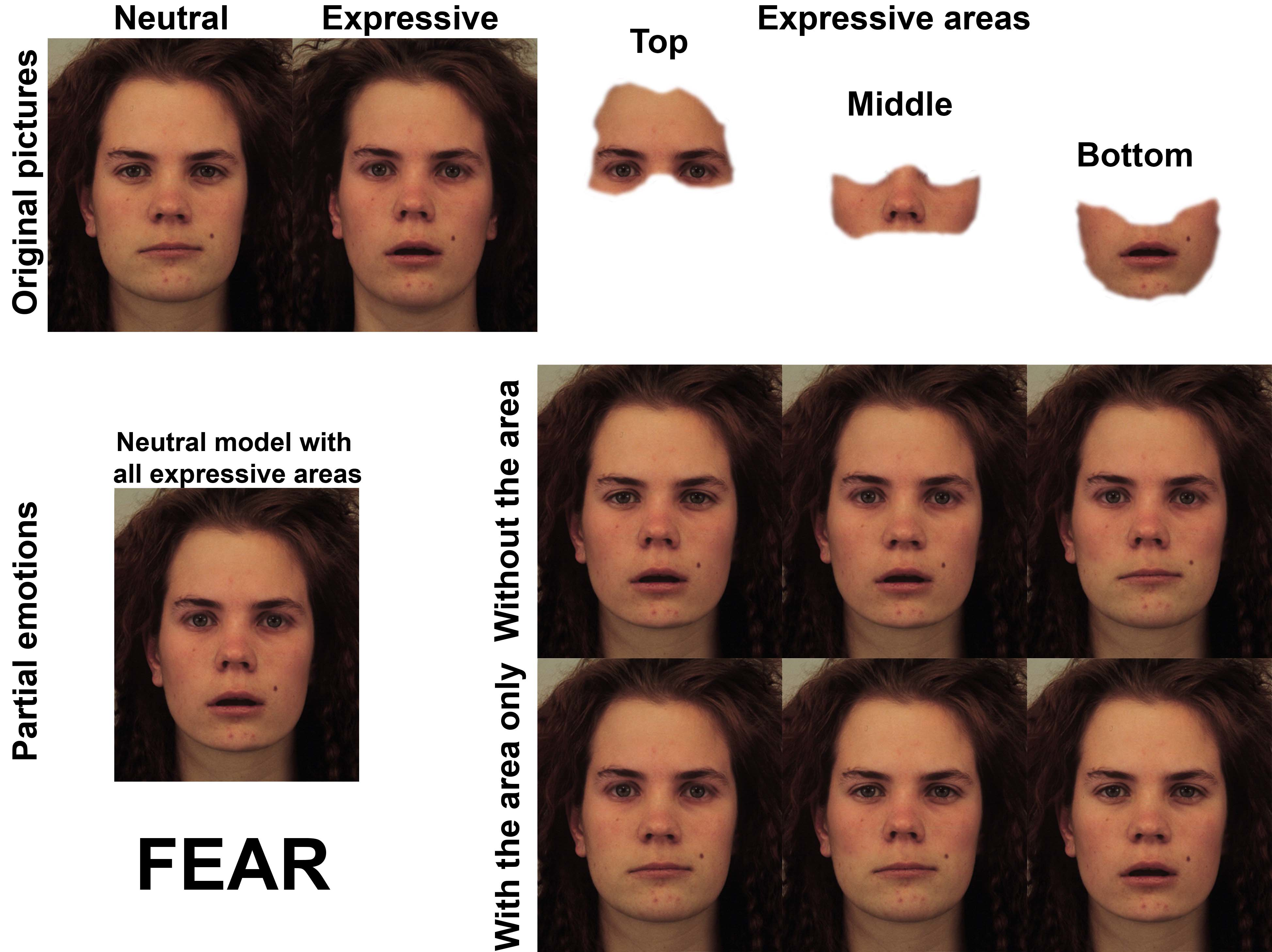

Supplement: S2 Fig — For each expression (top left), the top, middle and bottom parts were isolated (top right) and superimposed on the neutral expression of the same person to obtain a full expression (bottom left). Partial expressions were created either by removing one part from the full expression (bottom right, first row) or by superimposing only one part on the neutral face (bottom right, second row). (ZIP) [file pone.0245777.s002.zip › S2_Fig (fear partial).tif]

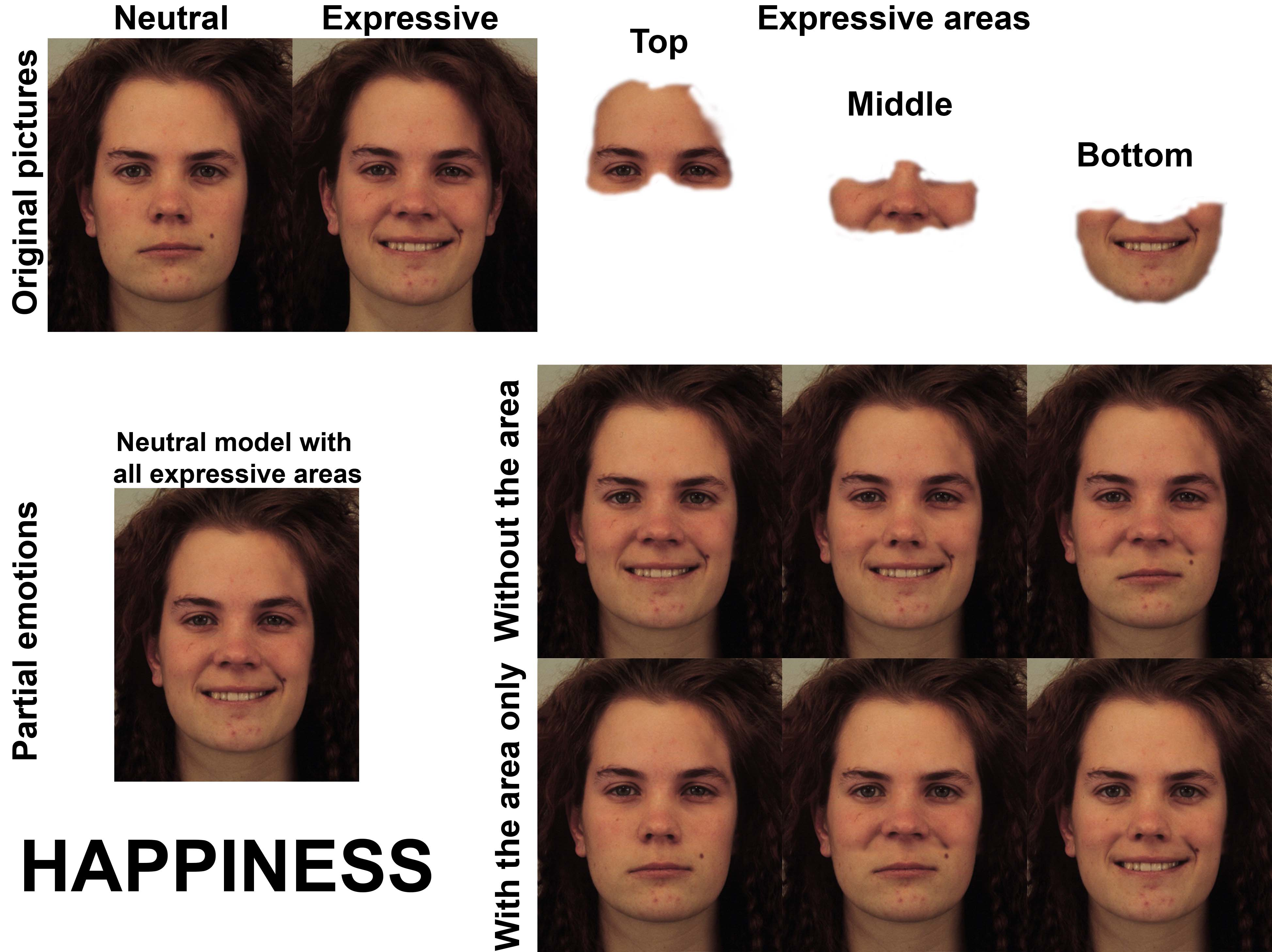

Supplement: S2 Fig — For each expression (top left), the top, middle and bottom parts were isolated (top right) and superimposed on the neutral expression of the same person to obtain a full expression (bottom left). Partial expressions were created either by removing one part from the full expression (bottom right, first row) or by superimposing only one part on the neutral face (bottom right, second row). (ZIP) [file pone.0245777.s002.zip › S2_Fig (happiness partial).tif]

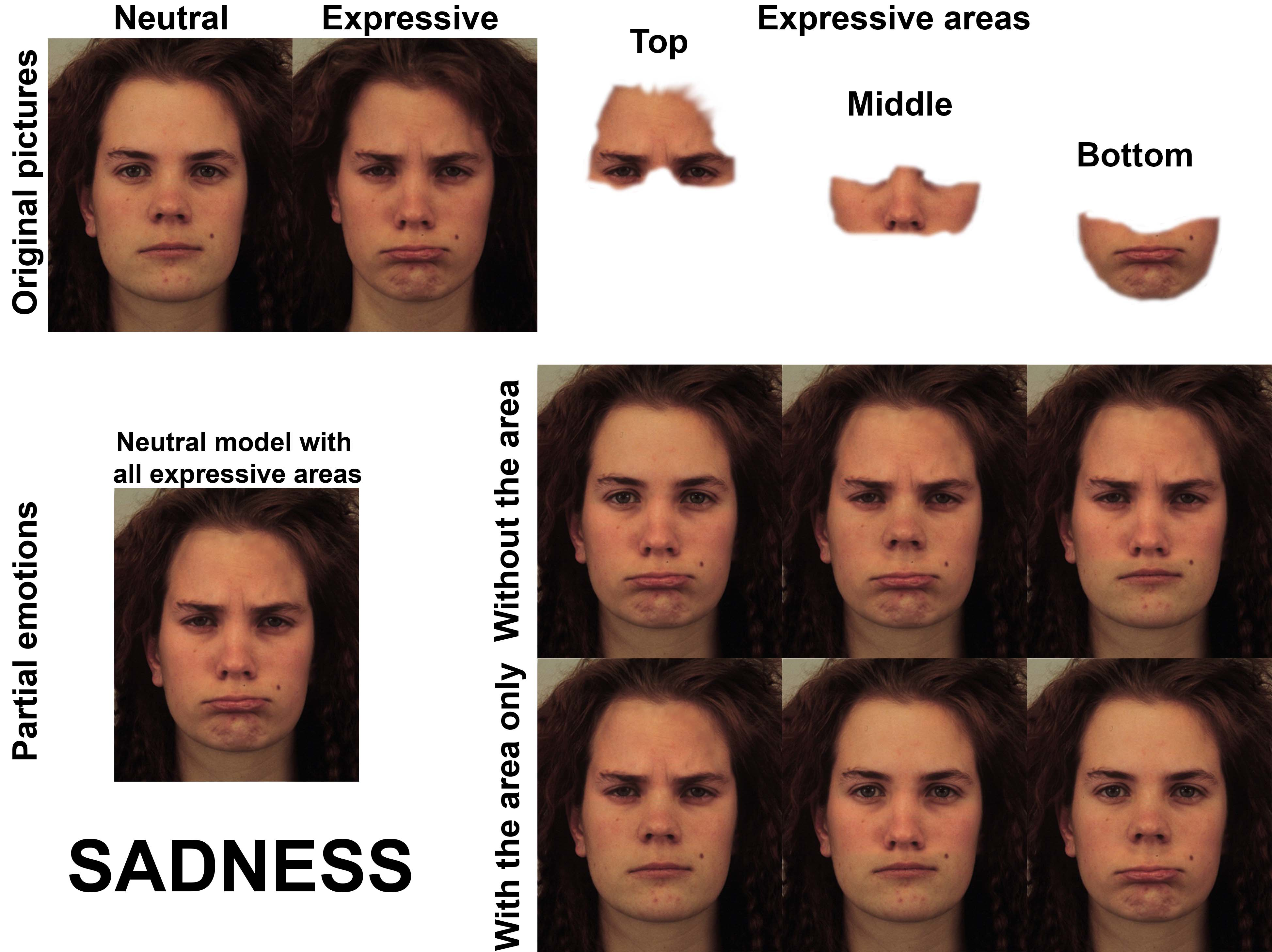

Supplement: S2 Fig — For each expression (top left), the top, middle and bottom parts were isolated (top right) and superimposed on the neutral expression of the same person to obtain a full expression (bottom left). Partial expressions were created either by removing one part from the full expression (bottom right, first row) or by superimposing only one part on the neutral face (bottom right, second row). (ZIP) [file pone.0245777.s002.zip › S2_Fig (sadness partial).tif]

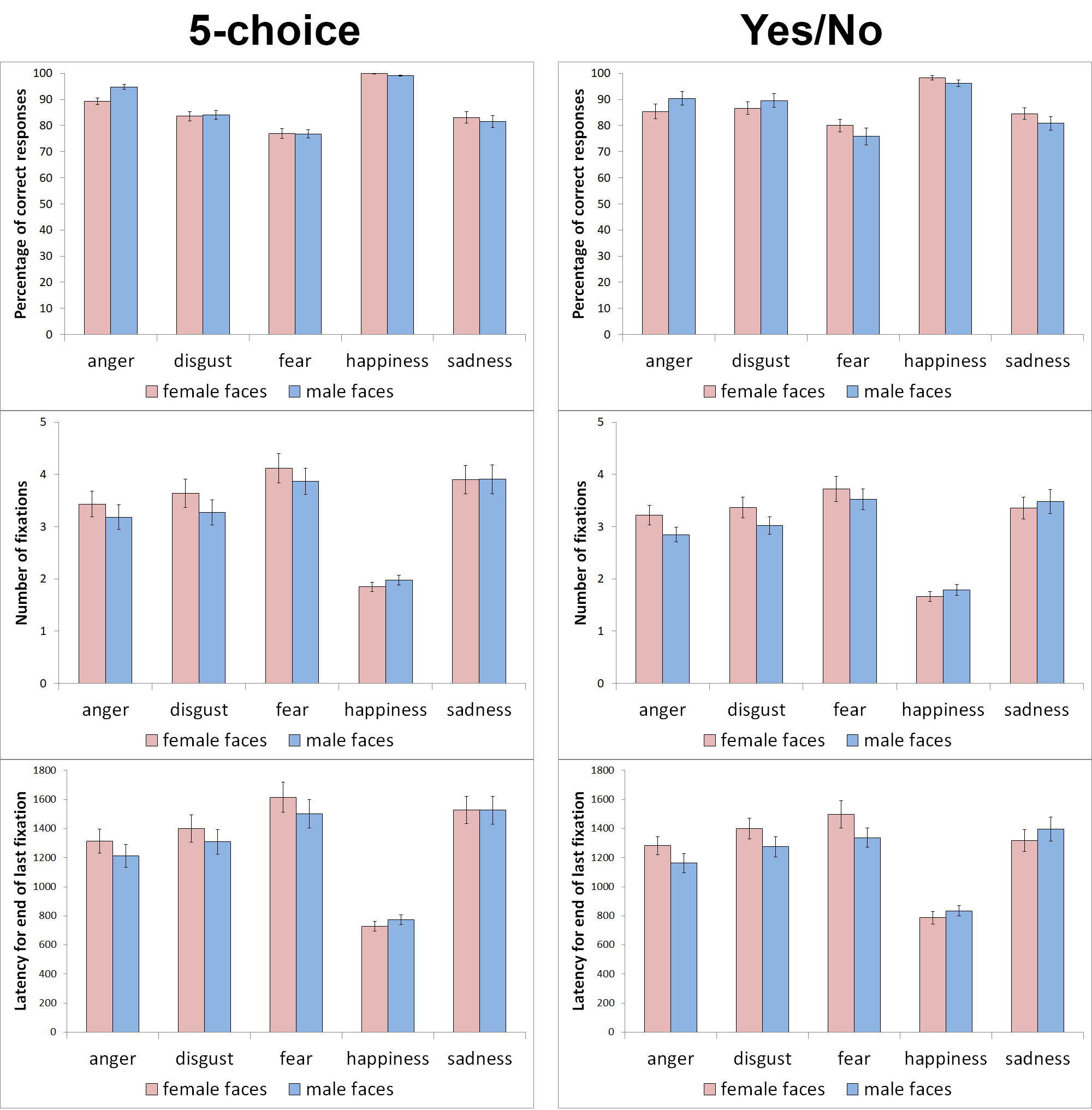

Supplement: S3 Fig — Error bars are SE. (TIF) [file pone.0245777.s003.tif]

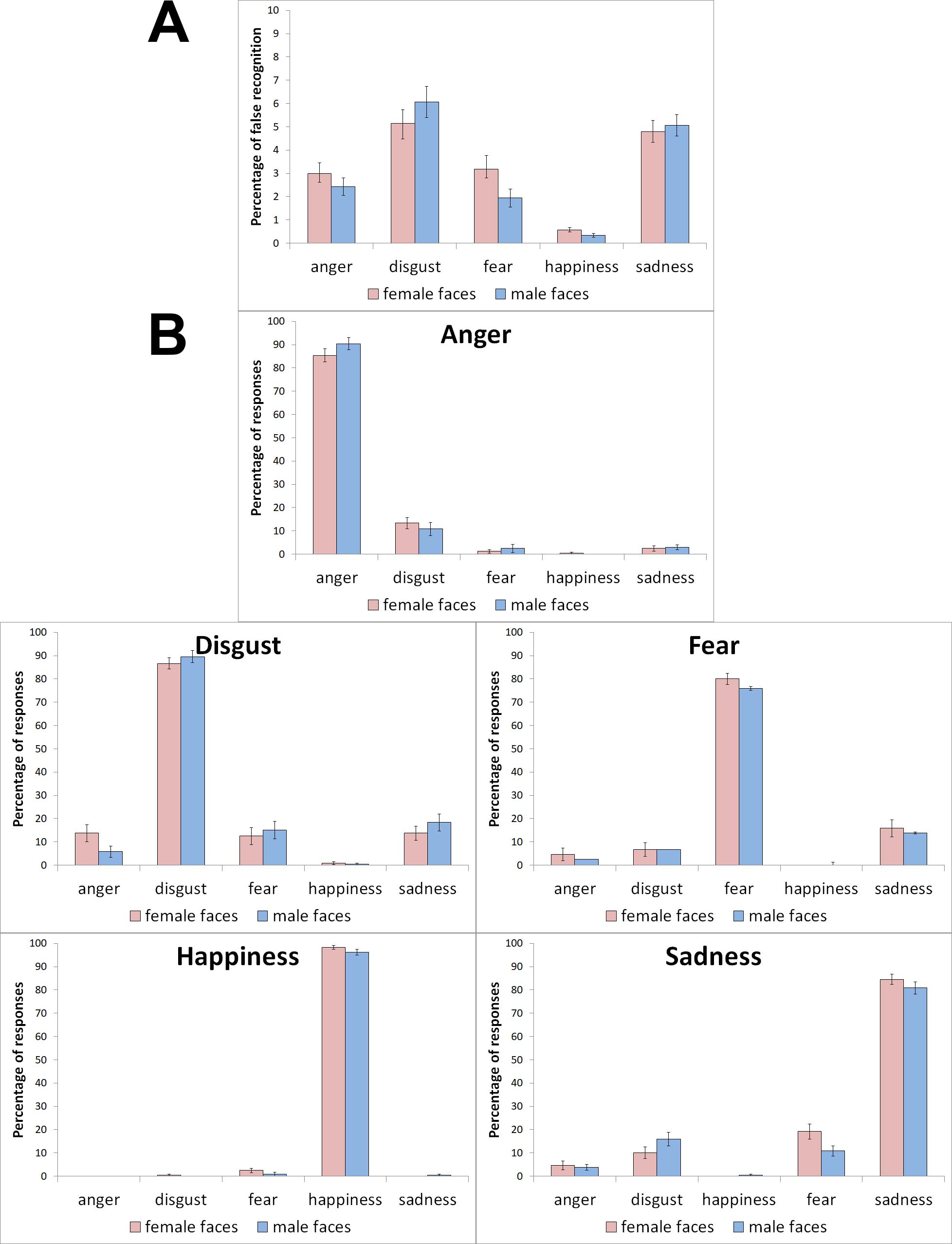

Supplement: S4 Fig — A) Percentage of time each emotion was perceived in a model that expressed another emotion, according to the emotion that was perceived and the gender of the model (e.g., for anger, percentage of time the decoders responded “anger” when the models simulated either disgust, fear, happiness, or sadness). B) Repartition of the responses (in %) for the five emotions expressed by the models, according to the response and model’s gender. Error bars are SE. (TIF) [file pone.0245777.s004.tif]

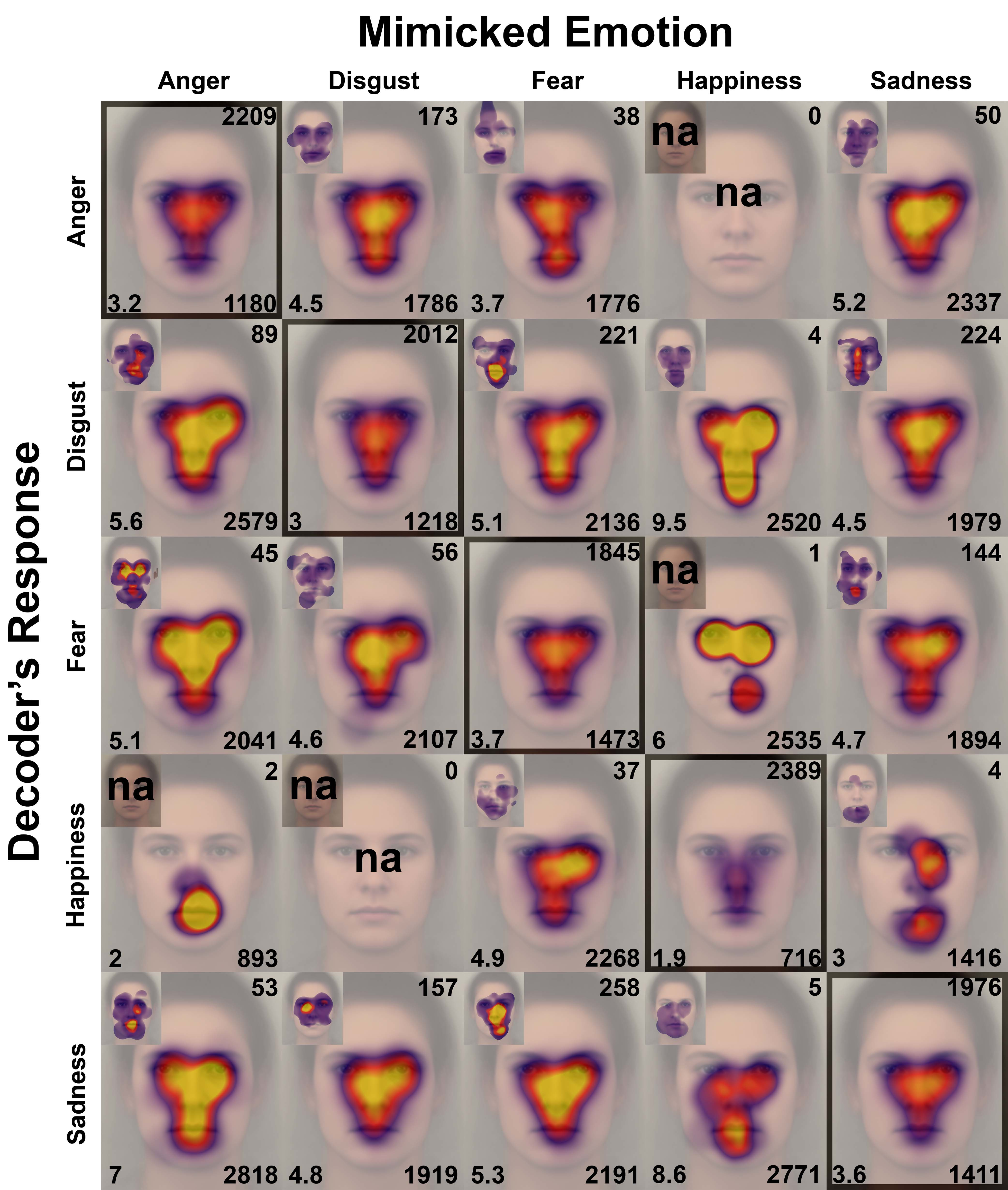

Supplement: S5 Fig — Mean time (large face) and T values (small face) according to the emotion the model attempted to mimic and the response of the decoder. The values at the corners of the faces indicate the number of such errors over the course of the experiment (top right), the number of fixations (bottom left) and the latency to the end of last fixation for a correct response (bottom right). na: not applicable. (TIF) [file pone.0245777.s005.tif]

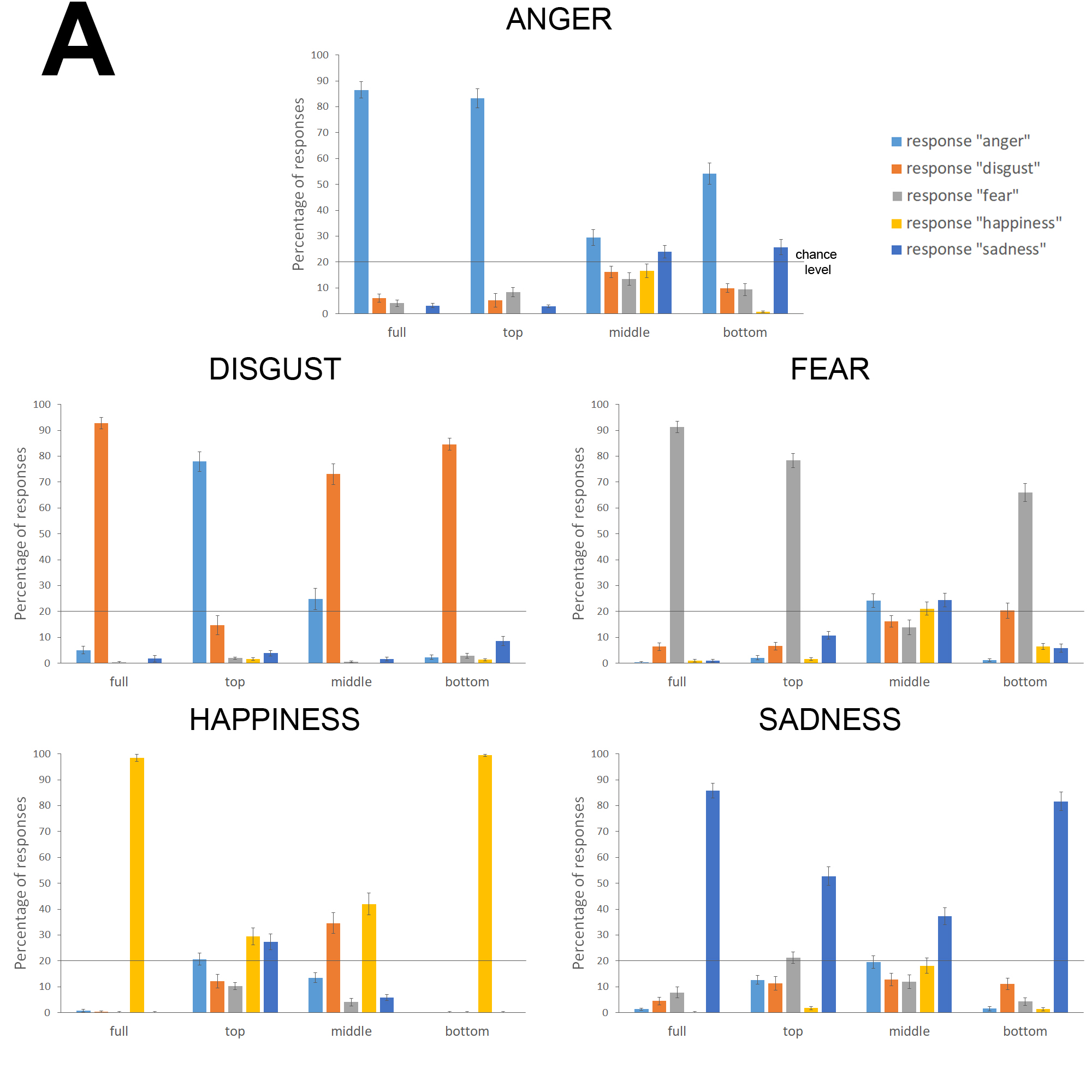

Supplement: S6 Fig — A) The percentage of time each emotion was perceived according to the emotion and the facial region that was removed. B) The percentage of time each emotion was perceived, according to the emotion and the facial region that was displayed alone. Error bars are SE. For the statistical comparison of correct responses in the different conditions, see Fig 4 in the manuscript. (ZIP) [file pone.0245777.s006.zip › S6A_Fig.tif]

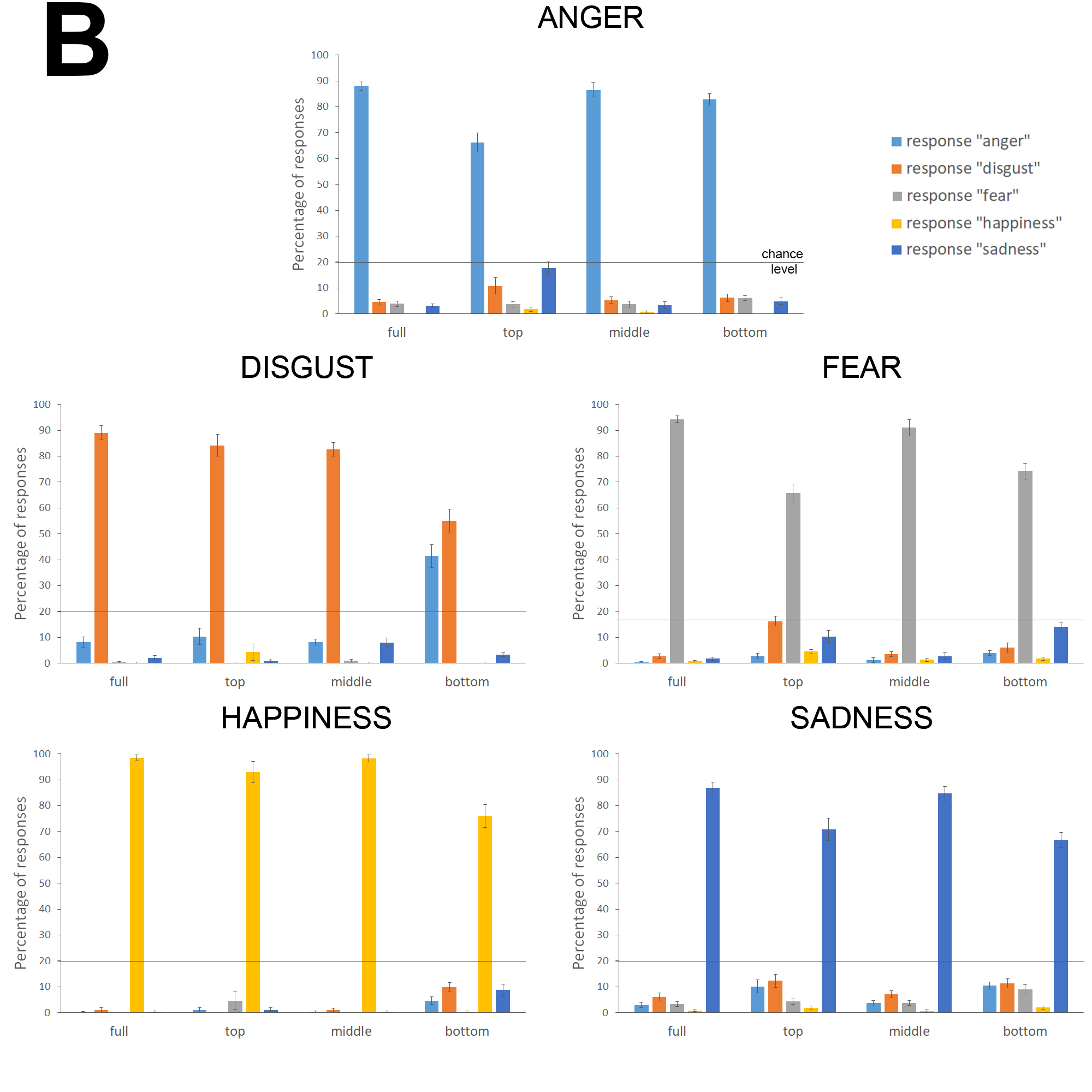

Supplement: S6 Fig — A) The percentage of time each emotion was perceived according to the emotion and the facial region that was removed. B) The percentage of time each emotion was perceived, according to the emotion and the facial region that was displayed alone. Error bars are SE. For the statistical comparison of correct responses in the different conditions, see Fig 4 in the manuscript. (ZIP) [file pone.0245777.s006.zip › S6B_Fig.tif]

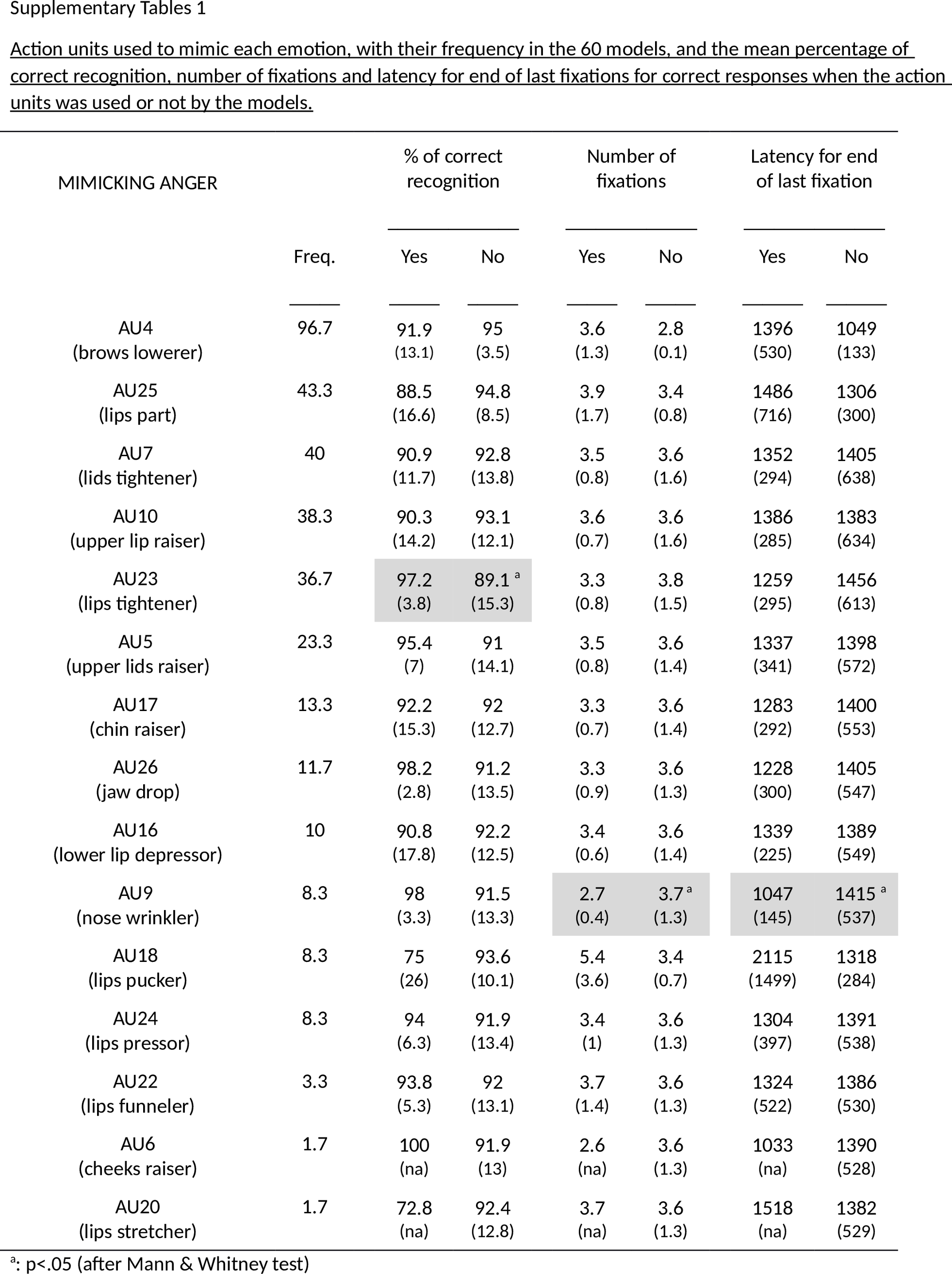

Supplement: S1 Table — (ZIP) [file pone.0245777.s007.zip › S1_Table (anger).tif]

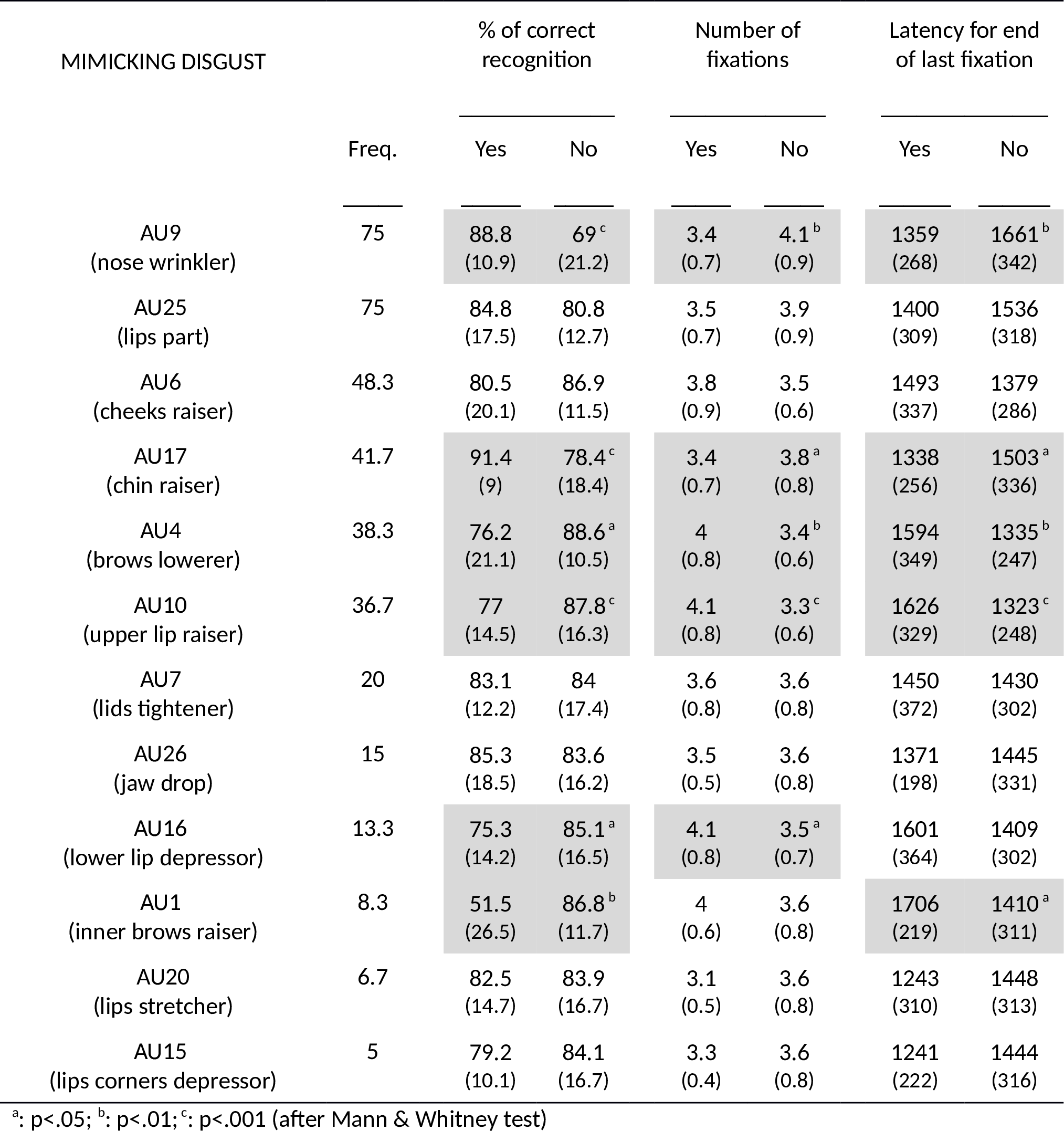

Supplement: S1 Table — (ZIP) [file pone.0245777.s007.zip › S1_Table (disgust).tif]

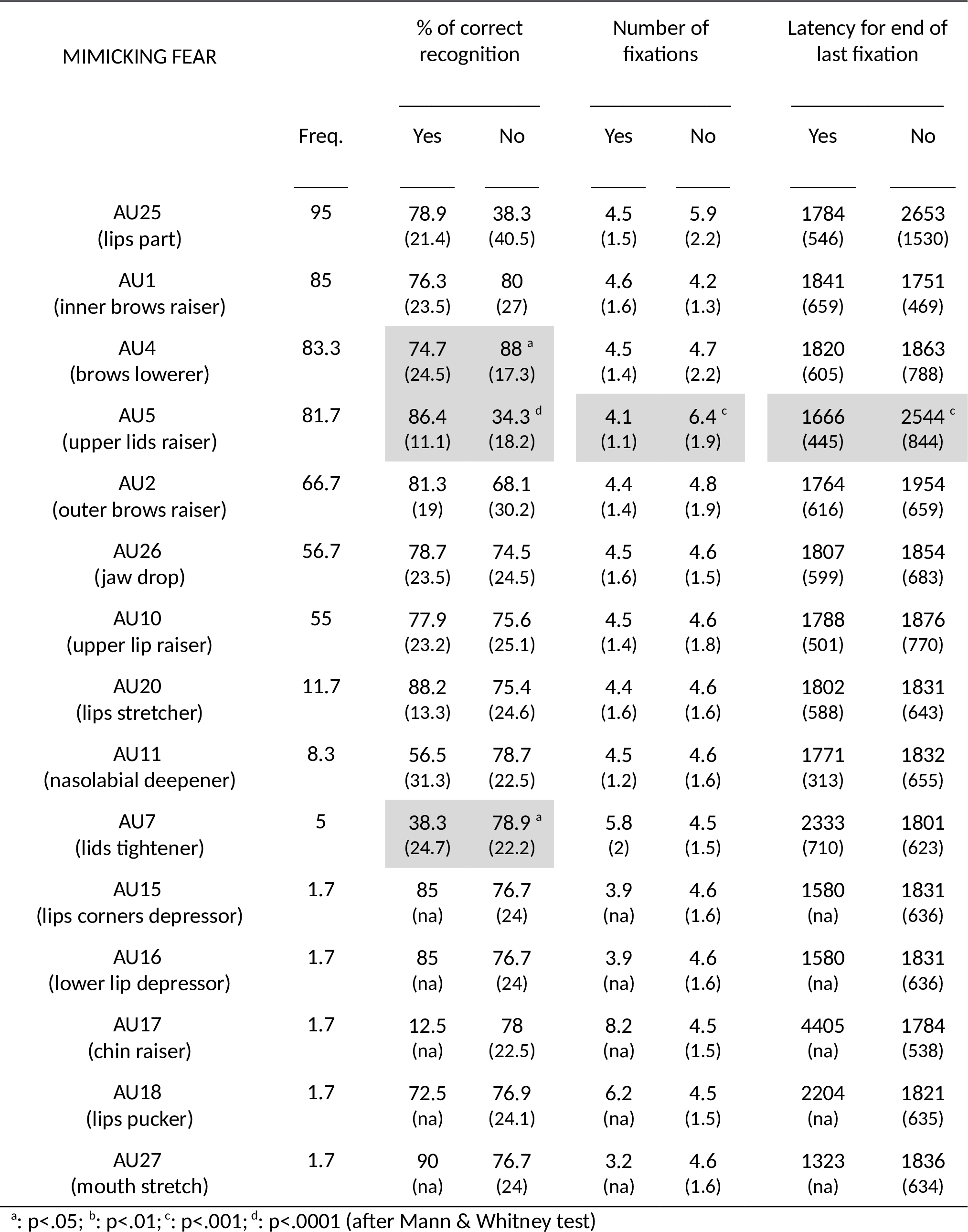

Supplement: S1 Table — (ZIP) [file pone.0245777.s007.zip › S1_Table (fear).tif]

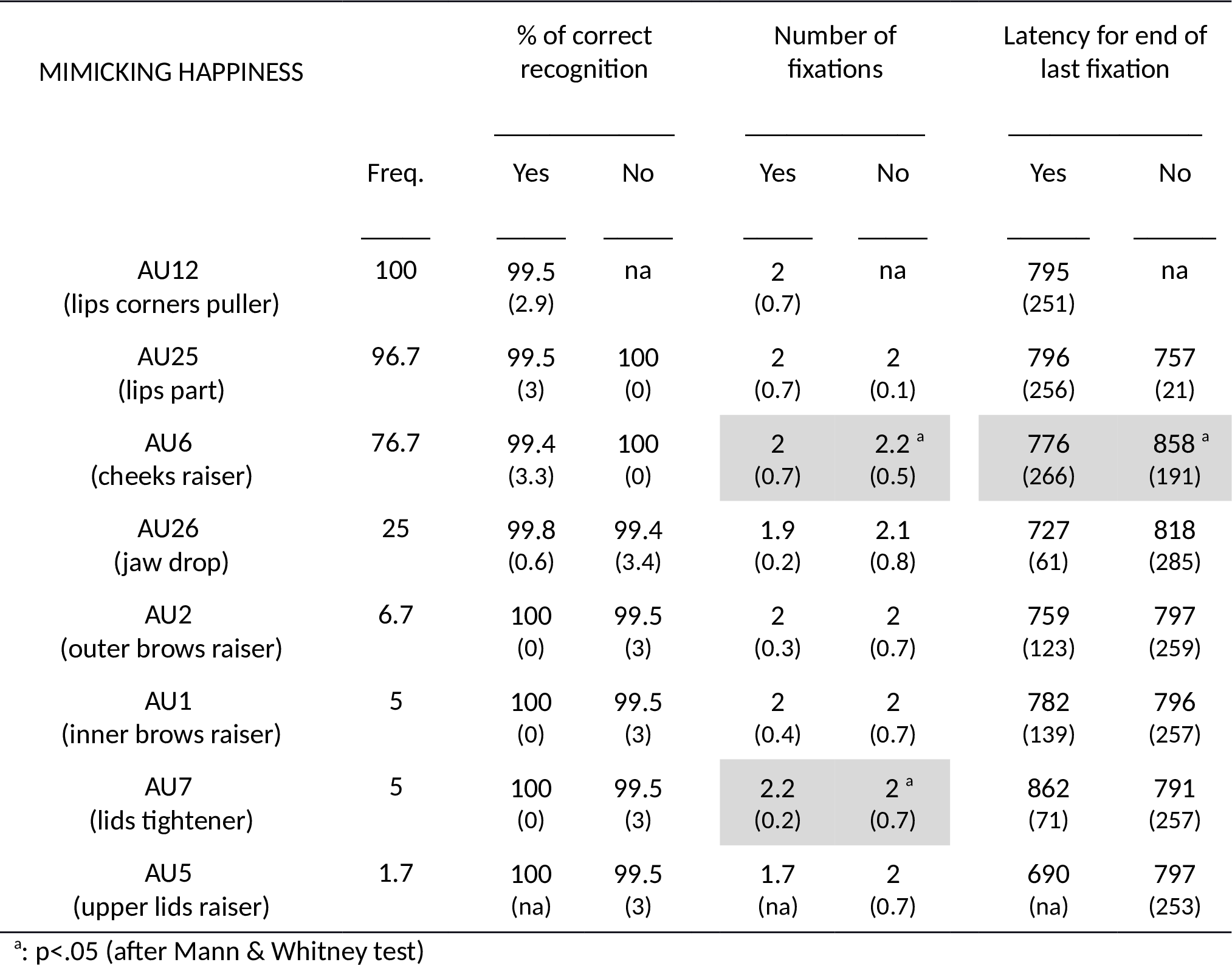

Supplement: S1 Table — (ZIP) [file pone.0245777.s007.zip › S1_Table (happiness).tif]

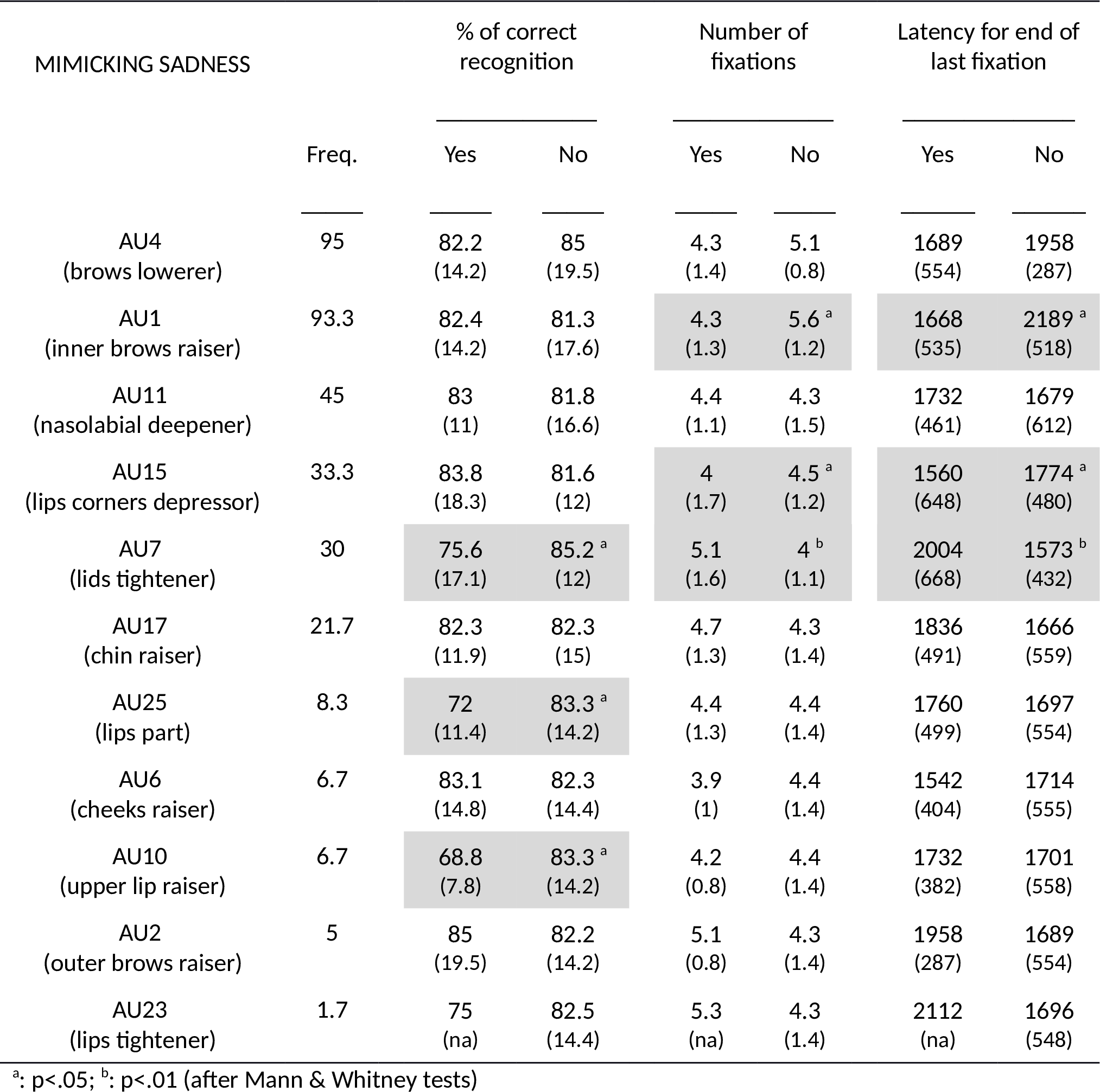

Supplement: S1 Table — (ZIP) [file pone.0245777.s007.zip › S1_Table (sadness).tif]
